# Human alpha defensin 5 is a candidate biomarker to delineate inflammatory bowel disease

**DOI:** 10.1371/journal.pone.0179710

**Published:** 2017-08-17

**Authors:** Amanda D. Williams, Olga Y. Korolkova, Amos M. Sakwe, Timothy M. Geiger, Samuel D. James, Roberta L. Muldoon, Alan J. Herline, J. Shawn Goodwin, Michael G. Izban, Mary K. Washington, Duane T. Smoot, Billy R. Ballard, Maria Gazouli, Amosy E. M'Koma

**Affiliations:** 1 Department of Microbiology and Immunology, Meharry Medical College School of Medicine, Nashville, Tennessee, United States of America; 2 Department of Biology, Lipscomb University, Nashville, Tennessee, United States of America; 3 Department of Biochemistry and Cancer Biology, Meharry Medical College School of Medicine, Nashville, Tennessee, United States of America; 4 School of Graduate Studies and Research, Meharry Medical College School of Medicine, Nashville, Tennessee, United States of America; 5 Department of Surgery, Vanderbilt University School of Medicine, Nashville, Tennessee, United States of America; 6 Vanderbilt-Ingram Cancer Center, Vanderbilt University School of Medicine, Nashville, Tennessee, United States of America; 7 Department of Pathology, Meharry Medical College School of Medicine, Nashville General Hospital, Nashville, Tennessee, United States of America; 8 Department of Pathology, Microbiology, and Immunology Tennessee Valley Health Systems VA Medical Center, Vanderbilt University Medical Center, Nashville, Tennessee, United States of America; 9 Department of Surgery, Augusta University Medical Center, Augusta, Georgia, United States of America; 10 Department of Pathology, Vanderbilt University School of Medicine, Nashville, Tennessee, United States of America; 11 Department of Medicine, Meharry Medical College School of Medicine, Nashville, Tennessee, United States of America; 12 Department of Basic Medical Sciences, Laboratory of Biology, Medical School, National and Kapodistrian University of Athens, Athens, Greece; National Cancer Institute, UNITED STATES

## Abstract

Inability to distinguish Crohn's colitis from ulcerative colitis leads to the diagnosis of indeterminate colitis. This greatly effects medical and surgical care of the patient because treatments for the two diseases vary. Approximately 30 percent of inflammatory bowel disease patients cannot be accurately diagnosed, increasing their risk of inappropriate treatment. We sought to determine whether transcriptomic patterns could be used to develop diagnostic biomarker(s) to delineate inflammatory bowel disease more accurately. Four patients groups were assessed via whole-transcriptome microarray, qPCR, Western blot, and immunohistochemistry for differential expression of Human α-Defensin-5. In addition, immunohistochemistry for Paneth cells and Lysozyme, a Paneth cell marker, was also performed. Aberrant expression of Human α-Defensin-5 levels using transcript, Western blot, and immunohistochemistry staining levels was significantly upregulated in Crohn's colitis, ***p< 0*.*0001***. Among patients with indeterminate colitis, Human α-Defensin-5 is a reliable differentiator with a positive predictive value of 96 percent. We also observed abundant ectopic crypt Paneth cells in all colectomy tissue samples of Crohn's colitis patients. In a retrospective study, we show that Human α-Defensin-5 could be used in indeterminate colitis patients to determine if they have either ulcerative colitis (low levels of Human α-Defensin-5) or Crohn's colitis (high levels of Human α-Defensin-5). Twenty of 67 patients (30 percent) who underwent restorative proctocolectomy for definitive ulcerative colitis were clinically changed to *de novo* Crohn's disease. These patients were profiled by Human α-Defensin-5 immunohistochemistry. All patients tested strongly positive. In addition, we observed by both hematoxylin and eosin and Lysozyme staining, a large number of ectopic Paneth cells in the colonic crypt of Crohn's colitis patient samples. Our experiments are the first to show that Human α-Defensin-5 is a potential candidate biomarker to molecularly differentiate Crohn's colitis from ulcerative colitis, to our knowledge. These data give us both a potential diagnostic marker in Human α-Defensin-5 and insight to develop future mechanistic studies to better understand crypt biology in Crohn's colitis.

## Introduction

Managing phenotypic outcomes of indeterminate colitis (IC), given its unpredictable clinical presentation and disease course, is challenging in endoscopic medicine [1,2]. Inadequate differentiated diagnostic features of the two predominantly colonic main inflammatory bowel disease (IBD), namely ulcerative colitis (UC) and Crohn’s colitis (CC), may lead instead to an inconclusive diagnosis of IC [1,2]. A significant subgroup of IBD patients are misdiagnosed or have diagnosis delayed even when a state-of-the-art classification system of clinical, endoscopic, radiologic and histologic tools is used [2–5]. Study strategy is to enhance accurate and noninvasive diagnostics technologies that can measure individual molecules in complex endoscopic and surgical clinical specimen and provide quantitative and qualitative data about cellular system and can differentiate pathologic from normal tissue to delineate diseases within the same organ [6,7,8].

In addition, in predominantly colonic IBD, the distinction between UC and CC is of utmost importance when determining a patient’s candidacy for restorative proctocolectomy [9–11]. Approximately 15% of patients whose IC is surgically treated with IPAA resolve with an ultimate diagnosis of *de novo* Crohn’s, for which surgery may have been contraindicated [1,12]. Construction of an ileoanal pouch in these patients indicate that positive outcomes require painstaking clinical evaluations to identify those with Crohn’s or IC that turns to *de novo* Crohn’*s*, which may result in pouch failure and significant morbidity in the interim [10,13]. Change of diagnosis of IC to UC or CC is sometimes observed in patients with IBD before colectomy, as is the development of *de novo* Crohn’s disease after surgery, restorative proctocolectomy (RPC) and ileal pouch-anal anastomosis (IPAA) [1,10,12,13,14]. Incorrect diagnosis and treatment carries potential morbidity from inappropriate and unnecessary surgeries and underscores the necessity of research efforts aimed at a more accurate diagnosis of the colitides [6,7,10,11]. This paper describes a potential diagnostic molecular biomarker for reclassifying IC into an accurate clinical diagnosis of UC or CC prior to appropriate care intervention. These successful studies are the first of their kind to use tissue molecular biometrics in the colon microenvironment to delineate the colitides [6,7].

In this study, we found that expression levels of HD5 were up to 118-fold greater in CC as compared to UC. HD5 is a human protein that is encoded by the *DEFA5* gene and is highly expressed in secretory granules of crypt Paneth cells (PCs) of the small intestine (ileum) [15]. Of note, crypt PCs are normally not found in normal colon epithelium [16]. However, we observed abundant PCs in all CC colectomy tissues and in one patient with UC, indicating that there may be stem cell niche factors in this tissue. Others had similar observation [16,17]. This study is the first to significantly extend what others have presented earlier about changes in PC density and morphology, based on lysozyme distribution in PCs of ileal Crohn’s/Crohn’s ileitis [18,19]. HD5 is a microbicide and cytotoxic peptide involved in host defense mechanisms and is responsible for non-specific killing of gut microbes [15]. To date, there is no available single molecular biomarker that has proven to have sufficient diagnostic qualities for IBD. In this study, we provide HD5 as a possible diagnostic candidate biomarker. HD5 is a more reliable signature to distinguish CC from UC. Developing a better diagnostic marker will allow patients previously diagnosed as IC and UC, but in reality were CC and received inappropriate surgical intervention, to receive recommended appropriate therapies.

## Methods

### Clinical samples and ethical consideration

In order to carryout tissue profiling of differentially expressed proteins/genes in IBD, we first sought ethical approval from the Meharry Medical College (IRB file #: 100916AM206) and Vanderbilt University Medical Center (IRB file #s: 080898 and 100581) Institutional Review Boards [20]. Informed consent was provided, and patient participation in the study was voluntary. Patient samples comprised of surgical pathology colectomy tissues from adults with definitive UC and CC phenotypes as well as those diagnosed with IC at Vanderbilt University Medical Center (VUMC) between 2000 and 2007. The full thickness surgical samples of colectomy tissue were analyzed by pathology teams at MMC and VUMC, Schools of Medicine following established protocol criteria for IBD subtypes. For each selected sample, medical records data on patient demographics, preoperative variables prior to and after time of ileal pouch-anal anastomosis surgery, surveillance endoscopic and clinical findings, and medical and surgical treatment history were reviewed retrospectively. Samples included in all experiments were taken from various parts of the colon; all inflamed tissue unless otherwise indicated. A condensed list of samples and colon locations are included in S1 Table.

### Diagnostic criteria for inflammatory bowel disease

Pathology teams at MMC and VUMC Schools of Medicine used the following protocol criteria for the final surgical pathology reporting.

#### For ulcerative colitis

Characteristic pattern of involvement of colon, worse distally in untreated patients; lack of perianal or fistulizing disease; no granulomas, except in association with ruptured/injured crypts; no transmural lymphoid aggregates or other transmural inflammation; no involvement of terminal ileum, except mild “backwash ileitis” in cases with severe cecal involvement and no pyloric metaplasia in terminal ileum.

#### For Crohn’s disease

Involvement of other sites in the gastrointestinal tract (skip lesions, segmental disease); perianal or fistulizing disease; granulomas, not in association with ruptured/injured crypts and terminal ileum involvement.

#### For indeterminate colitis

Distribution favors UC, but focal transmural inflammation, or inflammation in ileum more than expected in backwash ileitis and no fistulizing disease.

### Vanderbilt patient medical records database

The availability of a detailed IBD patient database registry at Vanderbilt University Medical Center (VUMC) made chart review and follow-up surveillance possible. Medical records data on patient demographics, preoperative variables prior to and after IPAA surgery, surveillance of endoscopic and clinical findings, and medical and surgical treatment history were retrieved retrospectively.

### Indeterminate colitis clinical retrospective study

A retrospective investigation was conducted to identify a cohort of patients diagnosed with IC and registered in the IBD Center at VUMC. Twenty-one patients, initially classified as IC at the time of diagnosis between years 2000–2007, were identified and reevaluated for disease course in 2014, after a mean surveillance follow-up of 8.7±3.7 (range, 4–14) years, in order to identify the rates of diagnosis resolution to UC or CC. Diagnosis for each patient was determined based on standard clinical and pathologic features as previously described [21,22]. Three gastrointestinal pathologists blinded to clinical diagnosis reconciled and confirmed colitis diagnosis for each patient and represented a consensus among treating physicians. Patients who clinically did not changed and maintained the IC diagnosis were tested via IHC and Nikon Element Advanced Research Analysis Software (NEARAS) for HD5 levels to determine if HD5 could be used to identify CC from UC.

### Restorative proctocolectomy operated patients’ retrospective study

One hundred twenty patients with definitive UC underwent RPC surgery between 4/18/2001 and 6/18/2008. Of the 120 patients, 67 had their diagnosis re-evaluated after a mean follow-up of 9.4 (range, 6–13) years of functionally acceptable pouches. Compiled medical records allowed us to re-evaluate a progressive course of UC patients following RPC. Clinical information needed for each of these patients was available in the IBD medical records registry database at VUMC. The aim was to reevaluate patients who underwent RPC operation for definitive UC and had a change in diagnosis to *de novo* Crohn’s ileitis. Patients who had a change in diagnosis should reconcile the molecular biometric test that delineates IC into CC; again using NEARAS for HD5 levels.

### cDNA microarray

We performed a whole-transcriptome microarray with RNA extracted and pooled from human full thickness colon samples from UC and CC patients (n = 5/group) (Affymetrix, Santa Clara, CA).

### NanoString nCounter human inflammation kit gene expression

RNA from UC and CCl tissue was processed by NanoString (NanoString Technologies Inc., Seattle, WA) to determine gene expression level according to the manufacturer protocol [23].

### Real-Time RT-PCR

Real-Time RT-PCR was used to measure transcript levels of HD5. RNA was extracted from three human colon biopsy samples each from moderate UC and CC, and diverticulitis (DV) as a non-IBD control (RNeasy Miniprep Kit, Qiagen, CA). cDNA was generated using iScript cDNA synthesis kit (Bio-Rad, Hercules, CA). Pre-designed TaqMan probes (Thermo Fisher Scientific, Waltham, MA) were purchased for HD5 and GAPHD control, and all samples were run in triplicate using a CFX96 qPCR thermocycler (Bio-Rad). Data were analyzed per the ΔΔCt method of analysis.

### Western blot and immunohistochemistry

Western blot was used to assess any differences in HD5 protein levels. Protein was extracted from a minimum of 10 colon biopsy samples each from mild, moderate, and severe UC; mild, moderate, and severe CC; and non-IBD DV control. Whole cell lysates were extracted from full-thickness colon samples using T-PER (Thermo Fisher Scientific) per manufacturer’s protocol. Bradford Assays (Bio-Rad) were run to determine protein concentration, and protein was loaded onto a 4–20% SDS-PAGE tris/glycine gel (Bio-Rad). Proteins were transferred to PVDF (Bio-Rad), and Western blots for HD5 and β-actin loading control were performed with primary and secondary antibodies (Santa Cruz, Dallas, TX) per manufacturer’s protocol. Blots were visualized with Opti-4CN colorimetric detection kit (Bio-Rad) and imaged with ChemiDoc XRS+ imaging system (Bio-Rad). Band intensities were measured and data analysis performed with Image Lab Software (Bio-Rad).

Five colon tissue protein extracts and staining of HD5 per disease by immunohistochemistry (IHC) was done as previously described.^24^ Quantification of HD5 staining was analyzed manually by microscopy and automatically quantified using Nikon's Eclipse Ti microscope with built-in NEARAS [24,25].

### NEARAS technology for quantification of immunohistochemistry staining

NEARAS (Melville, NY) was used to calculate the number of cells with HD5 staining in IHC tissue. A mean intensity threshold of 20 to 255 intensity units was established to eliminate a false-positive signal from background staining. A circularity parameter of 0.5 to 1 and equivalent diameter of 5–15 micrometer was used to select for cells. All threshold parameters were used in each image to count the number of HD5-positive cells in tissue samples.

### Statistical analysis

The Vanderbilt University Microarray Core Laboratory performed statistical analyses for the microarray. Transcriptome level fold changes and the significance of those changes were calculated using one way ANOVA with Bonferroni’s correction for multiple comparisons. Significantly changed transcripts were defined as having >2.0 fold expression change from controls and a Benjamini-Hochberg (BH) false discovery rate corrected ANOVA p-value <0.05.

All other statistical analyses were performed using GraphPad Prism v6 software [26]. qRT-PCR and IHC HD5 counts were examined by applying an unpaired two-tailed Student’s *t*-test with the Welch correction, respectively. Western blots were analyzed by ANOVA followed by Fisher’s test for multiple comparisons. Chi square tests were utilized for determining relatedness of HD5 levels to CC. For all statistical analyses, ***p< 0*.*05*** indicated a statistical significance.

### Dual staining of human α-defensin-5 and lysozyme

DoubleStain IHC was performed on a Lab Vision autostainer 360 (Thermo fisher) using Abcam’s M&R on human tissue (DAB & AP/Red) staining kit (ab210059, Abcam Biotechnology, Cambridge, UK). The manufacture’s recommended conditions were used with the following modifications. The mouse anti-α-defensin 5 (sc-53997, Santa Cruz Biotechnolgy, Inc, Dallas, TX) and rabbit anti-lysozyme (ab-2408) were used at a 1:50 dilution in OP Quanto antibody Diluent (Thermo Fisher, Waltham, WA). Prior to addition of antibody for 45 minutes, tissues were incubated for 10 min with Utravision hydrogen peroxide block (Thermo Fisher) followed by a 5 min incubation with Ultravisoion Quanto protein block. A single incubation with Permanent Red was used for ileum tissue, whereas two consecutive 10 min permanent Red incubations were performed for colonic tissue. Following hematoxylin counter staining, tissue was exposed to Richard-Allen Scientific Blueing Reagent (Thermo Fisher). Antigen retrieval was performed in 1 mM EDTA pH 8.4, 0.05% Tween 20 for 20 minutes at 98°C (60°C preheat/70°C cool down) using the Labvision PT Module (Thermo Scioentific). Image color deconvolution was performed with Fiji ImageJ 1.51f (http://imagej.nih.gov/ij) using the Fast Red, Fast Blue and DAB built in stain vector plugin.

## Results

### Nearly 30% of indeterminate colitis patients cannot be delineated into UC or CC

**Fig 1:**
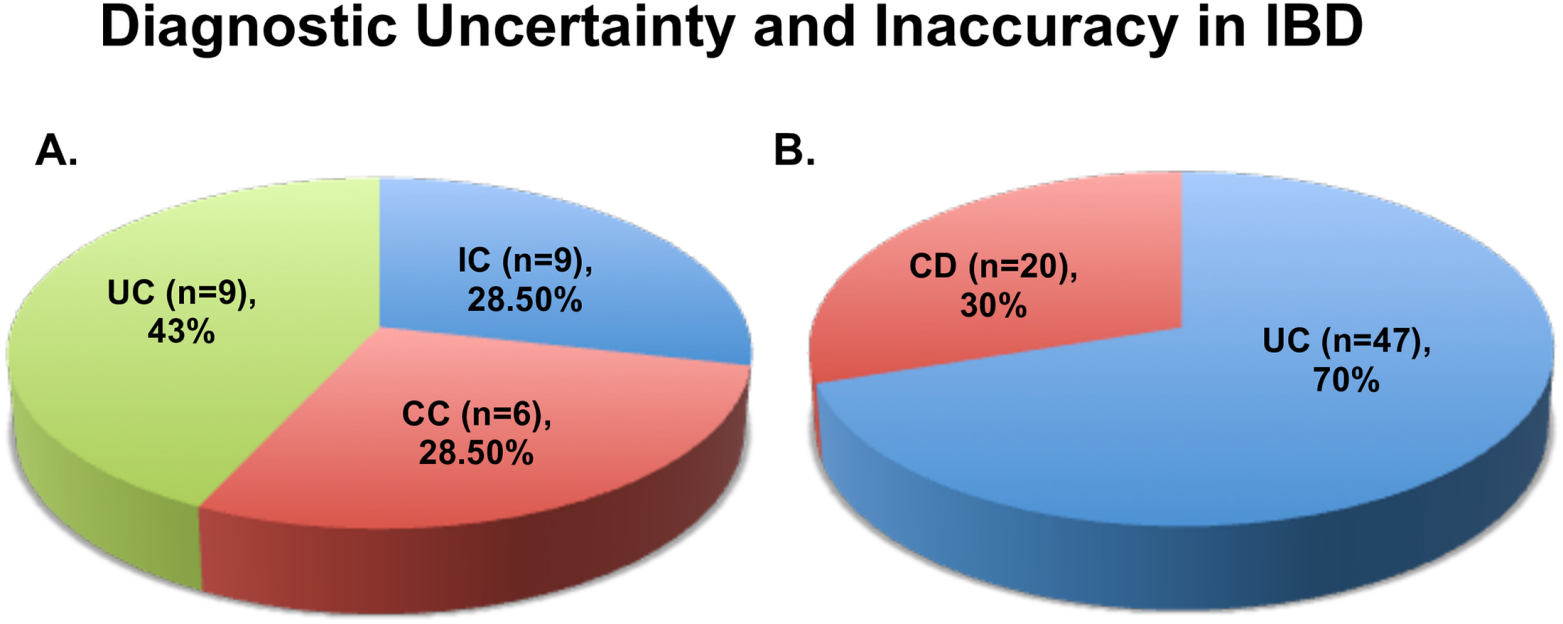
Diagnostic uncertainty and inaccuracy in IBD clinical setting. **A**, **Indeterminant colitis.** Twenty-one IC patients were followed for approximately ten years. At the end of the 10 year period, 28.5% of the patients could still not be delineated into a precise diagnosis of either UC or CC. **B**, **Crohn’s as Ulcerative colitis.** Sixty-seven UC RPC operated patients were followed for re-evaluated after a mean follow-up of 9.4 (range, 8–13) years for changing course of diagnosis. Thirty percent of these patients required a change of diagnosis to *de novo* Crohn’s disease.

A retrospective investigation was conducted to identify a cohort of patients diagnosed with IC to determine if they could be properly delineated into UC or CC over time. We followed 21 patients who were diagnosed with IC between the years 2000–2007 and reevaluated in 2014. A mean surveillance follow-up period was 8.7 ±3.7 (range, 4–14) years. Fifteen of the 21 (71.4%) had their original diagnosis changed; 9 to UC (43%) and 6 to CC (28.5%). Six (28.5%) patients remained clinically inconclusive and retained their diagnosis of IC (Fig 1A). These data were collected in the absence of any type of biomarker.

### Thirty percent of restorative proctocolectomy operated Crohn’s colitis patients were misdiagnosed as ulcerative colitis

A retrospective investigation was conducted to identify a cohort of patients that underwent RPC and IPAA surgery for a definitive UC diagnosis to determine if they had been misdiagnosed. We identified 67 such patients. A mean surveillance follow-up period was 9.4 (range, 6–13) years. A change in diagnosis to *de novo* Crohn’s disease of the ileal pouch was clinically observed in 20 (30%) patients (Fig 1B). In the other 47 (70%) cases, the initial diagnosis of UC remained clinically unchanged. These data were collected in the absence of any type of biomarker. Because of these results, we sought to determine if there are potential genes that may be used to better differentiate between UC and CC at first clinical biopsy and prior to any surgical intervention.

### There is differential expression of human α-defensin-5 in inflammatory bowel disease

We initially performed whole-transcriptome microarray with RNA extracted and pooled from human full thickness colon samples from UC and CC patients (n = 5) using the Affymetrix gene expression array according to the manufacturer’s instructions (Affymetrix, Santa Clara, CA) Tissues from diverticulitis (DV) were used as control. This analysis showed a total of 484 genes that were up- or down-regulated (≥ 2-fold) between the two diseases. Among the up-regulated genes were α-defensin-5, other antimicrobial peptides, and mucins (Table 1). HD5 was increased the most: 31-fold in CC *vs*. UC. A full list of the microarray results can be found in S2 Table.

**Table 1:** Abbreviated list of targets from affymetrix cDNA microarray. A total of 484 genes were highlighted in the microarray as potential markers for distinguishing UC from CC. The gene showing the largest fold change between the two diseases was Human Defensin 5 (HD5).

Gene Symbol	p-value (CC vs UC)	Fold Change (CC vs UC)
**DEFA5**	7.23E-05	31.0374
**REG1A**	0.00321456	21.9439
**MUC13**	0.0271079	4.11287
**MUC2**	0.01823535	3.26416
**LYZ**	0.0118582	2.39899

To replicate these data in a different platform, an independent analysis by PCR array (NanoString Technologies Inc. Seattle, WA) was carried out on 5 different human full thickness colon samples from UC and CC patients. Although the NanoString array only specifically targeted inflammatory genes, the only gene to show up in both the microarray and the PCR array was HD5. The NanoString array determined that HD5 was increased 118-fold in CC *vs*. UC in these human samples, compared to 31-fold in the previous samples analyzed by microarray (Table 2).

**Table 2:** Full list of targets from NanoString Human Inflammation PCR array. 16 inflammatory genes were changed in this subset of samples. HD5 was the only gene to appear in both the microarray and the NanoString PCR array.

Gene Symbol	NanoString p-value (CC vs UC)	NanoString Fold Change (CC vs UC)	Microarray Fold Change (CC vs UC)
**DEFA5**	0.00182525	118.145	31.0374
**RBP2**	0.282548	6.8909	--
**CD53**	0.417119	-1.32516	--
**SAA2**	0.575901	-1.36908	--
**SNORD13P2**	0.0839705	-1.42879	--
**SMAD4**	0.00233383	-1.49572	--
**SNORD28**	0.00995582	-1.58122	--
**ALOX5AP**	0.153036	-1.61452	--
**SCARNA8**	0.132287	-1.63997	--
**SNORD13**	0.00409278	-1.87394	--
**UNQ2550**	0.0386757	-1.97314	--
**CLEC4D**	0.168864	-2.03025	--
**STAP1**	0.211401	-2.03524	--
**CYP4F3LP**	0.0584598	-2.37697	--
**SAA1**	0.0988763	-2.42023	--
**IL6**	0.167391	-4.90534	--

**Fig 2:**
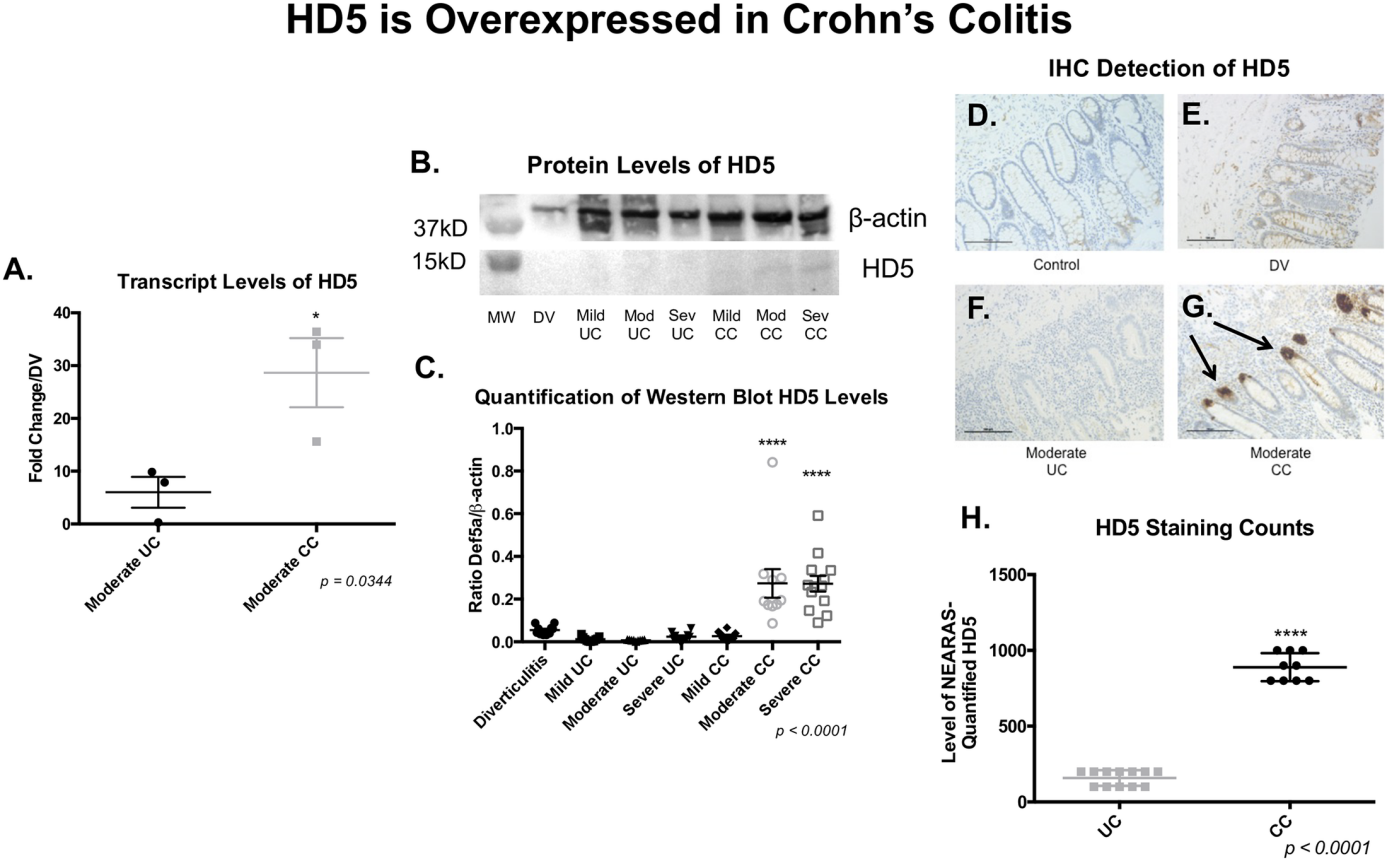
Aberrantly expressed of HD5 in IBD. **A**, Transcript levels of HD5 in moderate UC and CC. qRT-PCR confirms an increase in HD5 levels in moderate CC compared to moderate UC (*p<0*.*05*). Each point represents one patient sample. **B**, Representative HD5 Western Blot. HD5 levels (top) are higher in moderate and severe CC levels compared to all other disease states. β-actin loading control is shown on bottom. **C**, Graphical representation (densitometry) of HD5 Levels. Band intensities were measured for all samples and graphed as a ratio to β-actin loading control. Each point represents an individual sample. Moderate and severe CC levels of HD5 are both significantly higher than all other disease states (*p<0*.*0001*). **D-G** Representative IHC images of HD5 staining. **D**, Diverticulosis (DVL) no primary antibody control. **E**, Diverticulitis (DV). **F**, UC. **G**, CC (positive, arrow). **H**, HD5 staining counts. Each point represents one patient sample.

To further validate these data, we assessed the expression of HD5 by semi-quantitative RT-PCR using RNA extracted from moderate CC and moderate UC tissues (n = 3). This analysis also showed a significant increase in transcript levels of HD5 in CC compared to UC (Fig 2A, SEM, ***p< 0*.*05***). Several commercially available HD5 antibodies have been developed. Due to the sequence homology of the alpha defensin class of proteins, we tested a set of antibodies to assess specificity to HD5. We performed dot blots using commercially available antibodies against recombinant HD1-6. We determined that the monoclonal antibody from Santa Cruz Biotechnology, Inc. (Santa Cruz, CA) showed the highest level of specificity for HD5, and was therefore used in subsequent assays (S1 Fig). Next, we assessed the expression of HD5 by Western blotting (n≥ 10 for each disease state). Samples were run individually on western blots, with a combination of disease states on each blot, and we show an example of an individual sample per disease state in a representative blot (Fig 2B). When we take each individual sample into consideration across all western blots, protein densitometry analysis also shows significantly higher levels of HD5 in moderate and severe CC compared to all other disease states (Fig 2C, ***p< 0*.*0001***). Finally, we examined the expression of HD5 in moderate disease activity of IBD and control tissues by IHC using FFPE sections. This analysis revealed that HD5 levels are indeed increased in CC (Fig 2G) when compared to DV, and UC and normal (NL) control tissue (Fig 2D, 2E and 2F). Quantification of the HD5 IHC staining spot counts by NEARAS revealed a 5.6-fold increase of HD5 in CC *vs*. UC (Fig 2H, ***p< 0*.*0001***). We believe the IHC data explains the weak western blot banding patterns. Because the western blots were run with full-thickness samples, there is a low overall abundance of HD5 in the tissue; the IHC shows that it is much localized in the base of individual colonic crypts. Because of this, further analysis is done using IHC instead of western blots.

### Human α-defensin-5 levels are aberrant in indeterminate colitis and restorative proctocolectomy operated patients

All previous experiments indicate that HD5 levels are high in CC, but these samples were taken from patients who obtained a clear diagnosis of either UC or CC from the attending physician and pathologist. In order to determine if HD5 could be used to assess whether IC patients could be delineated into a diagnosis of either UC or CC, we assessed levels of HD5 in surgical pathology colectomy samples via IHC in patients described in Fig 1A. In each instance of a final diagnosis of CC, HD5 high NEARAS counts were in agreement with that diagnosis. We also found that when the 6 patients with unchanged IC diagnoses were analyzed via HD5 IHC NEARAS profile tests, 3 showed high HD5 count and agreed with the final diagnosis of CC (red font), and 3 low HD5 count and were in agreement with the final diagnosis of UC (green font) (Table 3).

**Table 3:** IHC staining for HD5 agrees with final diagnostic outcome in a sample of IC patients even when there was no agreement with the attending physician.

Patient Sample ID	Attending Pathologist Years 2000–2007	Attending Physician Years 2000–2007	Patient Outcomes Year 2014 New Diagnosis	Mean Area Fraction of HD5 (%) Count by NEARAS
**12-07-A1588**	IC	UC	UC	++
**12-10-A051A**	IC	IC	CC	++++++++
**ED56738T-003** **2^nd^ Opinion**	IC IC	UC UC	UC	+
**ED59253T-003** **2^nd^ Opinion**	IC IC	UC UC	UC	++
**M1121715A1**	IC	IC	UC	++
**M1122098A2**	IC	UC	UC	+
**M3124384A1**	IC	CC	UC	+
**M3124405A2** **2^nd^ Opinion**	IC IC	CC UC	CC	++++++++
**D-24672**	IC	UC	CC	++++++++
**D-4632**	IC	UC	UC	++
**D-3163**	IC	CC	CC	+++++++++
D-26455	IC	IC	CC	++++++++
D-26452	IC	CC	CC	++++++++
D-325	IC	UC	UC	+
D-2462	IC	CC	CC	++++++++++
A-24057	IC	UC	UC	++
A-24066	IC	CC	CC	++++++++++
A-24042	IC	IC	UC	++
56738T	IC	IC	CC	++++++++++
MAD12-625	IC	IC	UC	+
**M1151537AA**	IC	UC	UC	++

**Fig 3:**
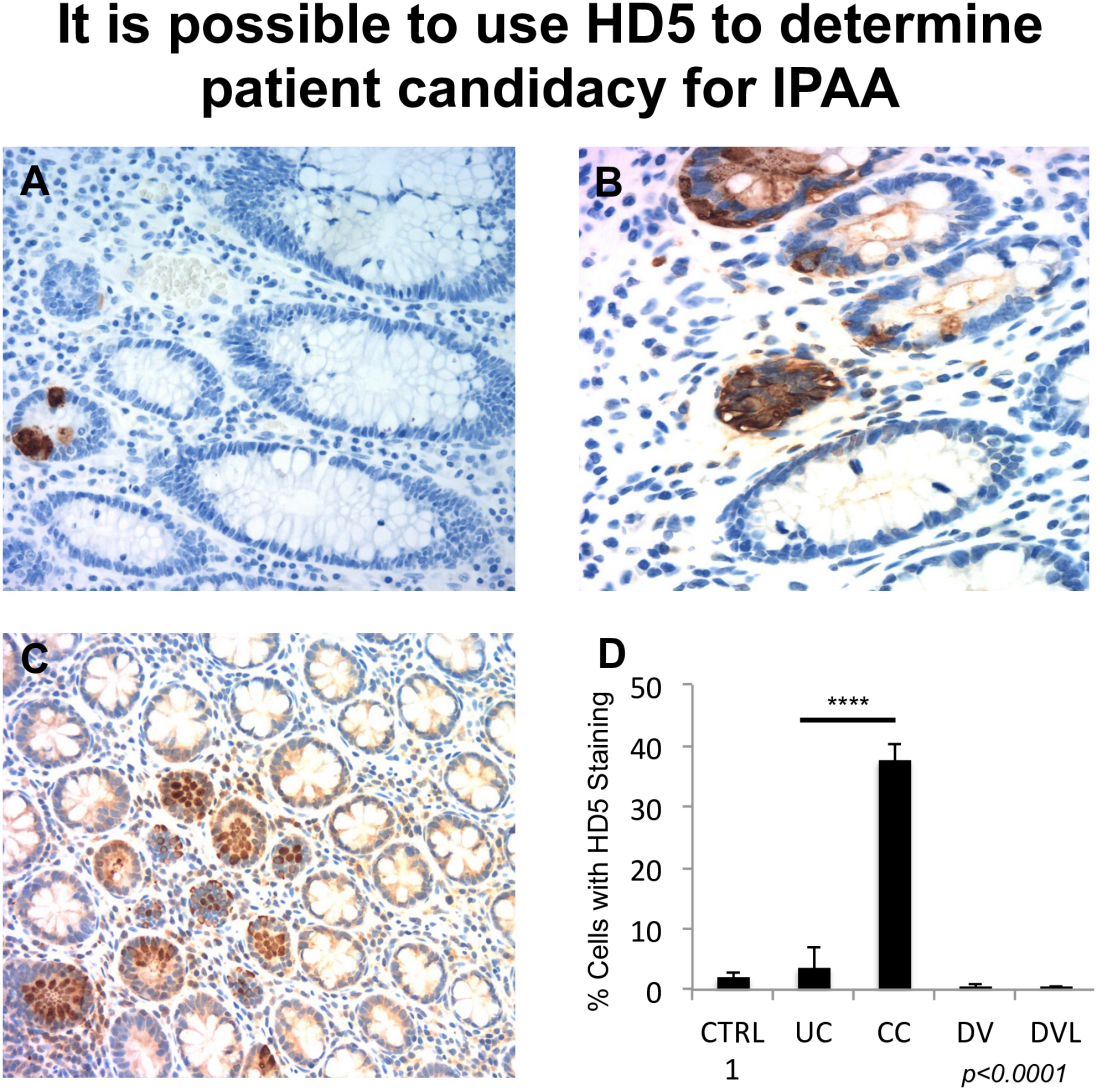
It is possible to use HD5 to determine patient candidacy for IPAA. A. Representative results from a RPC-operated patient that did not change the diagnosis after surgery and was molecularly tested using HD5 IHC. **B**, Representative results from a UC RPC and IPAA operated patients that did change the diagnosis from UC to *de novo* Crohn’s was molecularly tested using HD5 IHC. **C. NL-Ileum, control. D.,** Quantification of NEARAS HD5 IHC staining spot counts for UC RPC and IPAA-operated patients who did not have their original diagnosis changed *versus* those who did change from UC to *de novo* Crohn's (Fig 2A vs. 2B). (Ctrl 1 —staining control, UC—Ulcerative Colitis, CC—Crohn’s Colitis, DV—Diverticulitis, DVL—Diverticulosis).

Further, RPC and IPAA-operated patients described in Fig 1B who had a clinical change in diagnosis to *de novo* Crohn’s (n = 20) and those whose diagnoses did not change (n = 47) were also analyzed molecularly for HD5 levels via NEARAS IHC counts. The patients whose diagnosis remained unchanged showed only trace levels of HD5 (Fig 3A). Patients whose diagnoses clinically changed from UC to *de novo* Crohn’s showed prominent HD5 staining (Fig 3B). These images can be compared to normal ileum control (Fig 3C). Differential quantification of HD5 levels by NEARAS counts for UC RPC and IPAA-operated patients who did not have their original diagnoses changed *vs*. those with *de novo* Crohn’s (Fig 3A vs. 3B) were statistically significant (***p<0*.*0001***) (Fig 3D). In addition, statistical analysis to determine positive predictive values (PPVs) of HD5 in patient tissue are 95.8% for CC and only 76.9% for UC. Chi squared analysis shows significant relatedness between high levels of HD5 and a diagnosis of CC (***p<0*.*0001***). These data indicate that HD5 could be developed into a diagnostic tool to better distinguish CC from UC.

### Aberrantly regulated human α-defensin-5 in Crohn’s colitis patients may be caused by ectopic colonic crypt Paneth cells

**Fig 4:**
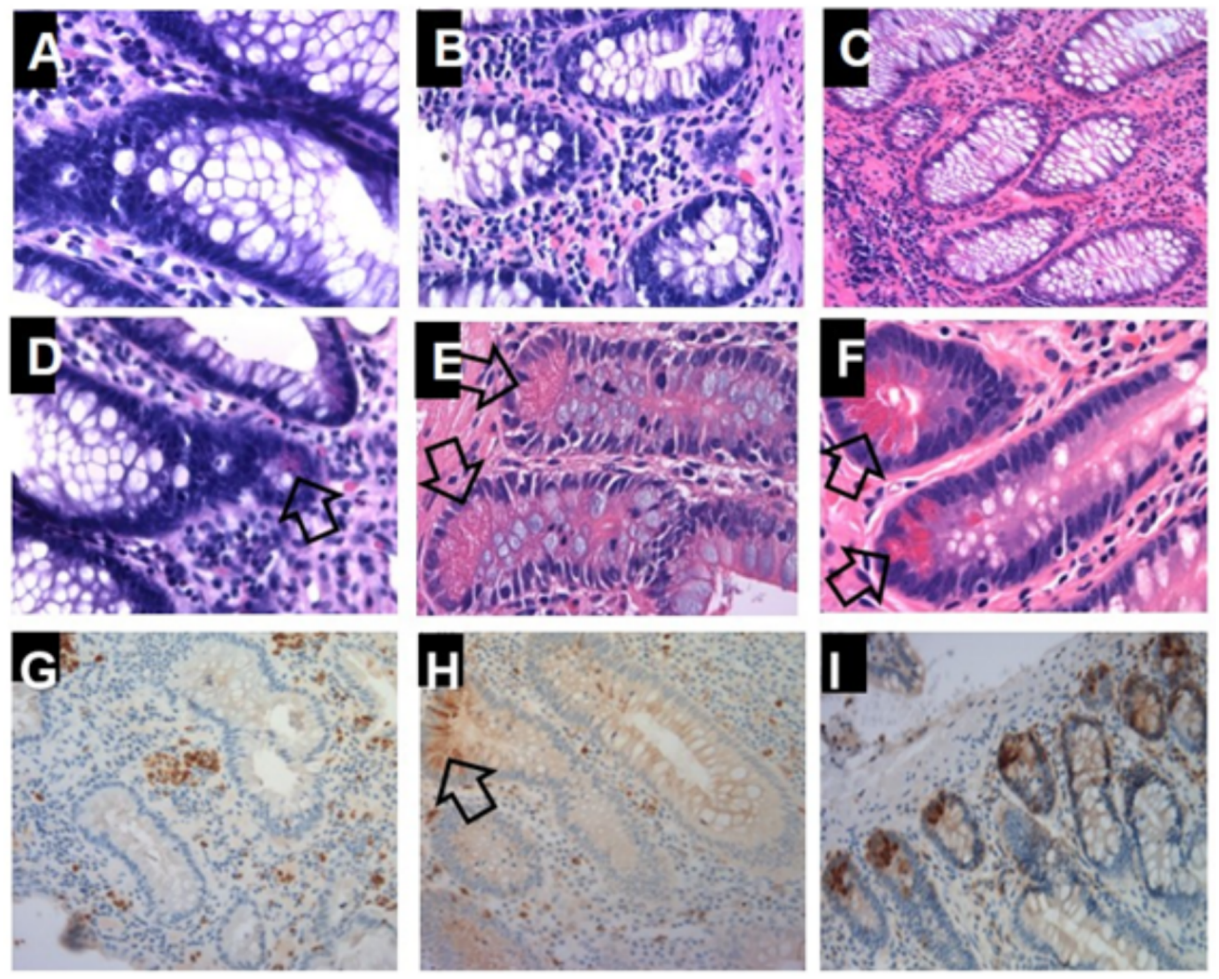
H&E staining on parallel sections the typical morphological appearance of Paneth cell (PCs) including the presence of dense apical eosinophilic granules. ***Upper panel:*** **A**, Diverticulitis (DV, no PCs), **B**, Diverticulosis (DVL, no PCs), **C**, Normal (NL-Colon, Control, no PCs). ***Middle panel*:** **D**, UC (found prodromal PC in one patient, arrow). **E**, CC, demonstrate abundance of PCs allover colonic basal crypts (arrows). **F**, Normal (NL-Ileum, Control), with abundance of PCs. ***Lower panel*:** **IHC detection of Paneth cell markers α-defensin 5 (DEFA5) and lysozyme (LYZ) in the colon. G**, NL-Colon, **H**, CC, and **I**, NL-Ileum, Control.

**Fig 5:**
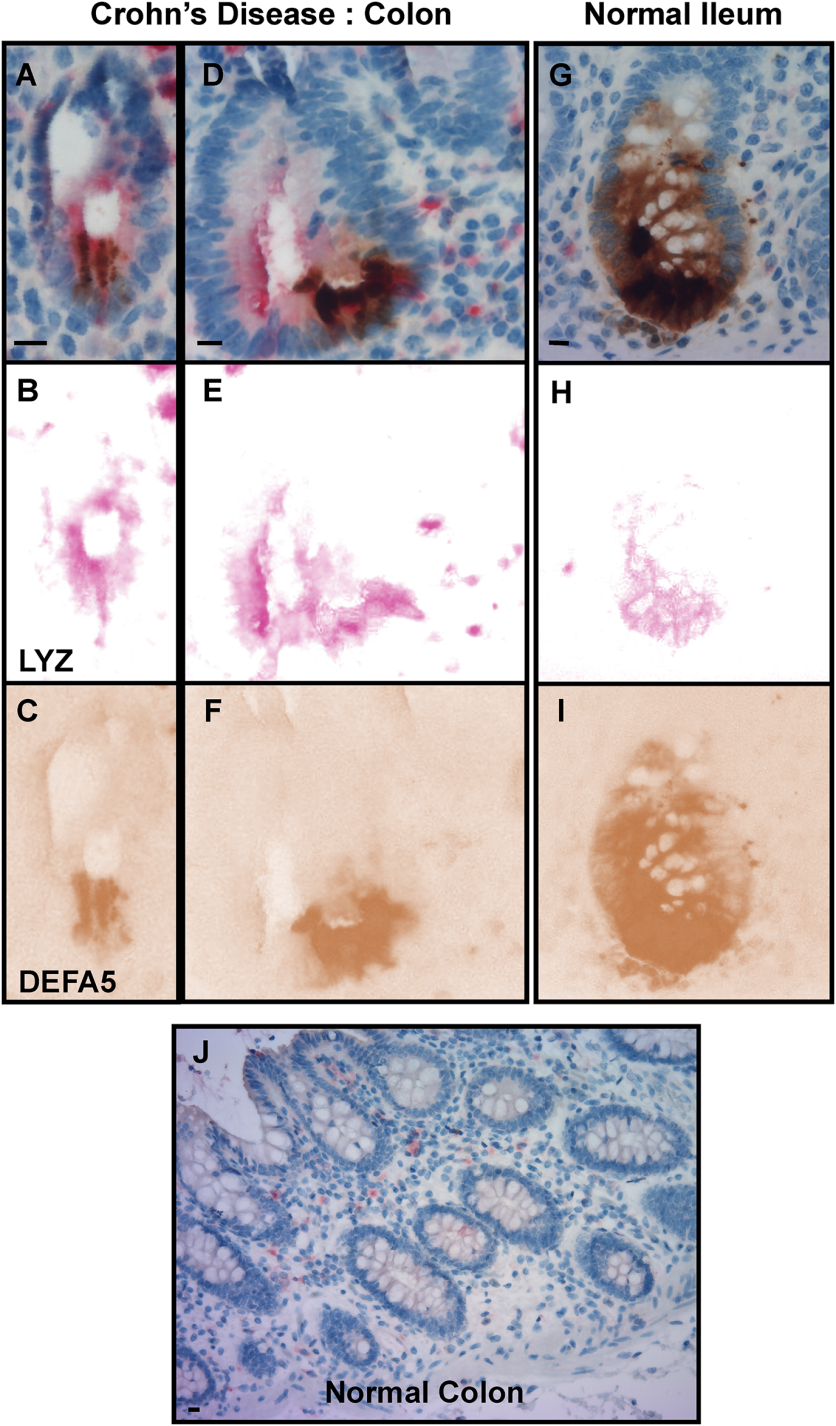
Double stain of PCs, lyzosomes and HD5. Double staining analyses from *de novo* Crohn’s (**Fig. 5A** and **D**), and normal ileum/control (**Fig. 5G**) are presented. Image deconvolutions are displayed vertically to evaluate lysozyme-specific permanent red (**Fig. 5B, E** and **H**) and HD5α-specific DAB (**Fig. 5C, F** and **I**). The normal colon image (**Fig. 5J**), which lacks PCs, was not further processed.

HD5 is a Paneth cell product; therefore, we wanted to determine if Paneth cells were present in the colon crypt of Crohn’s colitis patients. All 20 UC RPC operated patients with *de novo* Crohn's showed pools of ectopic crypt PCs in the colectomy samples, as demonstrated by H&E representative photomicrography (Fig 4E). This was validated by IHC labeling of PCs using lysozyme by microscopy, which confirmed the abundant presence of PCs in CC colonic crypts (Fig 4H, arrow). To validate whether the pool of HD5 expressed in CC and in *de novo* Crohn’s colectomy samples was indeed coming from colonic epithelial crypt PCs, we used immunohistochemically detection of PC markers α-Defensin 5 (DEFA5) and lysozyme (LYZ) and double staining IHC to colocalize PCs and HD5 on colectomy samples. Lysozyme alone detects PCs. We demonstrate the presence of abundant crypt PCs in CC colectomy patients (Fig 4H) compared to all other colonic conditions analyzed (UC, DV, DVL and NL). Further, double staining analyses from *de novo* Crohn’s (Fig 5A and 5D), normal colon (Fig 5J) and normal-ileum/control (Fig 5G) are presented. Image deconvolutions are displayed vertically to evaluate lysozyme-specific permanent red (Fig 5B, 5E and 5H) and HD5-specific DAB (Fig 5C, 5F and 5I). The normal colon image (Fig 5J), which lacks PCs, was not further processed. The results reconcile and represent a consensus among treating physicians.

### Human α-defensin-5 is a better candidate biomarker than Paneth Cells for Crohn’s colitis.

**Fig 6:**
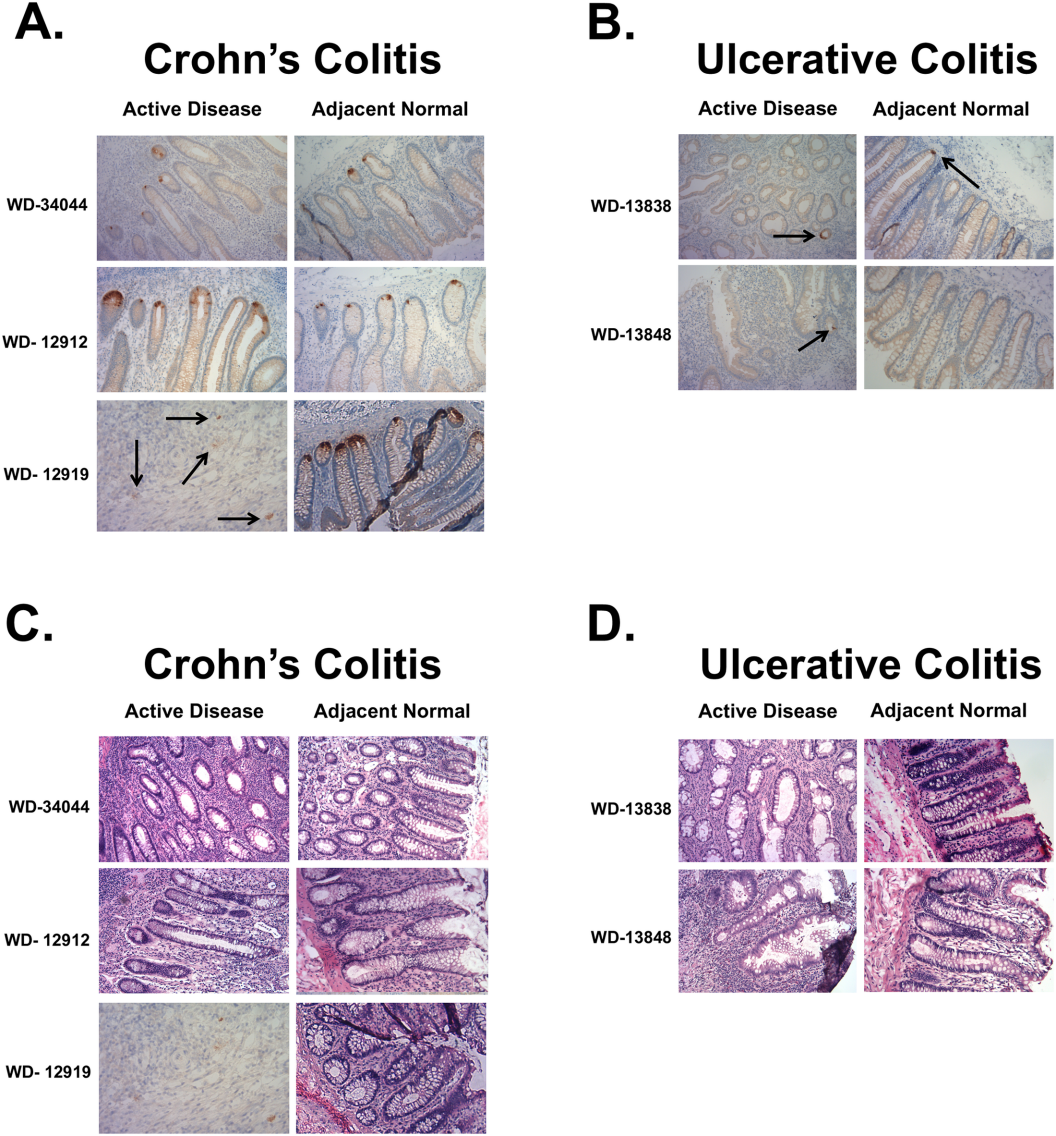
Assessment of HD5 and Paneth cells in inflamed and normal, adjacent tissue. HD5 staining of CC inflamed and normal, adjacent tissue shows expression of HD5 in all patient samples examined (**Fig. 6A**), compared to inflamed and adjacent, normal tissue of UC patients (**Fig. 6B**). H&E stains for Paneth Cells (**Fig. 6C and D**), were negative for PCs in all tissues.

Finally, we sought to determine if HD5 and Paneth cells were both upregulated in the normal, adjacent tissue of CC patients compared to UC patients (Fig 6). Immunohistochemistry for HD5 shows positive staining in the base of the crypts in both inflamed and normal, adjacent tissue of CC patient samples (Fig 6A). We were even able to see some positive HD5 staining when the crypt structure is abolished due to excessive inflammation and tissue damage (Fig 6A, patient WD-12919, arrows). Unsurprisingly, in UC tissue, we saw either very low levels of HD5 or no expression at all (Fig 6B). We were very surprised, however, to find that we could not see any Paneth cells in either the inflamed or normal adjacent tissues of any of the CC or UC patients surveyed (CC, n = 3; UC, n = 2) (Fig 6C and 6D). These results are surprising, considering our earlier experiments surveying Paneth cells in a larger number of patients (Fig 4). Because we can detect HD5 in the normal, adjacent tissue more readily than visualize Paneth cells, we believe that HD5 will serve as a better candidate biomarker than Paneth cells for CC.

## Discussion

We analyzed colectomy surgical pathology samples of patients with unambiguous CC and UC undergoing colectomy in connection with RPC and IPAA [6,7]. We identified and compared those protein profiles which had the necessary (i) specificity; (ii) sensitivity; (iii) discriminatory; and (iv) predictive capacity to determine the heterogeneity of IBD [6,7]. We were able to molecularly delineate UC and CC with molecular signatures of HD5 using IHC and quantified by NEARAS. Alpha-Defensins HD5 and HD6 are PC products and their altered expression has been linked to IBD pathogenesis.

Our Paneth cell data is interesting, and further work needs to be done to determine the role of Paneth cells in CC, specifically. Because HD5 is a Paneth cell product, we were surprised that we could not visualize PCs in these tissues even though we could detect HD5 in the same tissue (Fig 6), especially compared to earlier experiments showing high levels of PCs in all CC patients surveyed (Fig 4). In future experiments, we want to determine what cellular pathways are activated in the stem cells of the CC crypt, and if the levels of inflammation and/or severity of disease is important to when and how PCs appear in CC. These experiments are in progress. Whether the PCs are essential for stem cell maintenance *in vivo* remains debatable [27].

To date, there is no diagnostic gold standard tool for IBD. Differentiating UC and CC among patients with IC has remained painstaking and is a major challenge in endoscopic medicine and colorectal surgery [1,12,28,29]. Clinicians use an inexact classification system of clinical, endoscopy, radiologic, and histopathology findings in order to diagnose CC and UC [21,30,31]. Even with a combination of these diagnostic modalities, up to 15% of IBD patients are labeled as IC when no definitive evaluations can be made [13,30,32]. In addition, CC is mistakenly diagnosed and RPC and IPAA-operated as definitive UC in another 15% of IBD patients because of overlap in the clinical, endoscopic, radiological and histologic findings [12,33–36]. Further, most IC patients who undergo RPC and IPAA surgery for presumed UC are subsequently found to develop a recurrent *de novo* Crohn’s disease in the ileal pouch [1,12,33]. This is a serious consequence that may hinder the restoration of intestinal continuity and its intractable nature leads to pouch failure, often requiring pouch diversion or excision with a permanent terminal-ileostomy, resulting in negative psycho-sociological implications and poorer quality of life [1,29,31,36–41]. Curative treatment for UC is often surgical [42]. Success of RPC and IPAA surgery is largely dependent on careful patient selection combined with meticulous surgical technique and diagnostic accuracy [9–11,13]. Available clinical presentations and experience suggest that it is difficult to identify patients with CC who are likely to have a successful outcome after RPC and IPAA surgery [10,13,34,43]. However, in highly selected patients with CC, RPC and IPAA has been indicated [44–47]. If we are able to develop HD5 into a molecular marker, it may ultimately be used to select those patients with CC who are potential candidates for this sphincter preserving operation. Thus, RPC operation may be considered and should remain a careful option for certain subgroup of patients with CC, but an acceptable care option for patients with UC and for those IC patients predicted to develop UC [9,42].

Our studies of HD5 as a candidate biomarker for CC suggest it could be a diagnostic signature to efficiently distinguish CC from UC. Newly published data shows that patients with small bowel Crohn's disease (Crohn’s ileitis) are characterized with a deficiency of HD5, as shown by a reduced expression and secretion of the Paneth cell HD5, a fundamental feature of Crohn’s ileitis [48–51]. Our data, in CC, the reverse is true. We found Paneth cell HD5 to be a predominantly expressed antimicrobial peptide. This indicates that definitive CC and Crohn’s ileitis may have distinct etiologies and mechanisms. In these studies, we have reconciled all IC patient samples into UC and CC using molecular biomarker, HD5, and verified the reconciliation by patient outcomes (Fig 1 and Table 3).

Accurately distinguising CC from UC is of utmost importance when determining the candidacy of a patient for RPC [1,42]. Early diagnostic accuracy of IBD will lead to timely appropriate medical options. We have identified that HD5 can differentiate CC and UC and reclassify IC into CC. In addition to distinguishing the colitides, HD5 could objectively be used to evaluate biophysiological processes and therapeutic outcomes and potentially play a pivotal role in IBD clinics as an attractive, non-invasive avenue [52,53].

## Supporting information

S1 Table Biopsy locations. Various full-thickness human biopsy samples were utilized to perform the experiments. This table shows the sample locations that were utilized in each patient group.
(TIF)

S2 Table Complete list of microarray targets. A total of 484 genes were shown to be altered significantly +/- 2-fold between UC and CC according to the Affymetrix Microarray. All genes with p<0.05 are included in this table.
(PDF)

S1 Fig Antibody specificity assay. Dot blots were performed on recombinant HD1-6 with various commercial antibodies to determine specificity to HD5. Ponceau S Stain is used as a loading control. We found that the antibody from Santa Cruz was the most specific for HD5 of those tested.
(TIF)
