# Correction: Human alpha defensin 5 is a candidate biomarker to delineate inflammatory bowel disease

**DOI:** 10.1371/journal.pone.0189551

**Published:** 2017-12-06

**Authors:** Amanda D. Williams, Olga Y. Korolkova, Amos M. Sakwe, Timothy M. Geiger, Samuel D. James, Roberta L. Muldoon, Alan J. Herline, J. Shawn Goodwin, Michael G. Izban, Mary K. Washington, Duane T. Smoot, Billy R. Ballard, Maria Gazouli, Amosy E. M’Koma

In Fig 6, the “Active Disease” image for WD- 12919 in Fig 6A was incorrectly duplicated in Fig 6C. Please see the correct Fig 6 here.

**Fig 6:**
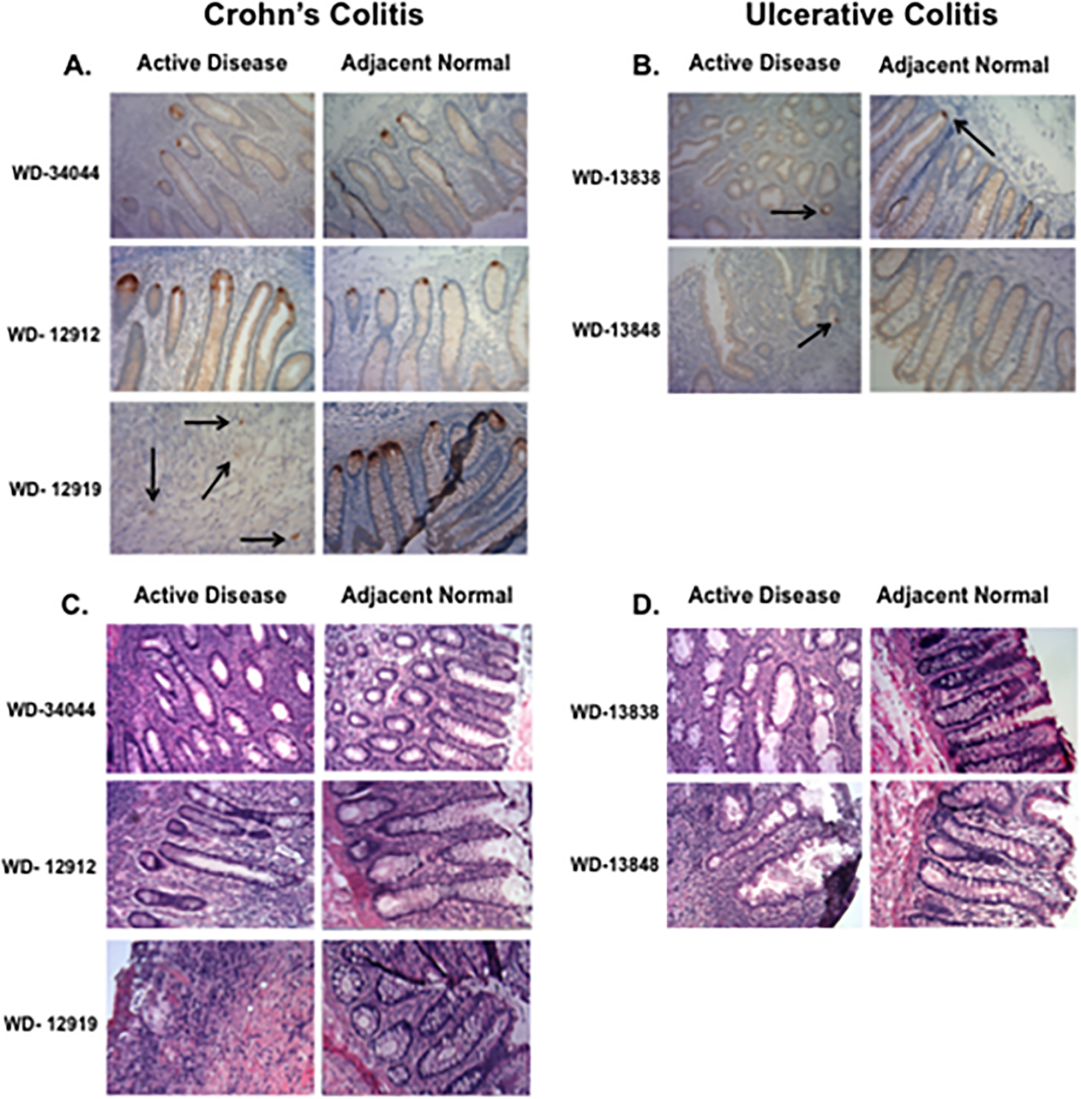
Assessment of HD5 and Paneth cells in inflamed and normal, adjacent tissue. HD5 staining of CC inflamed and normal, adjacent tissue shows expression of HD5 in all patient samples examined (**Fig. 6A**), compared to inflamed and adjacent, normal tissue of UC patients (**Fig. 6B**). H&E stains for Paneth Cells (**Fig. 6C and D**), were negative for PCs in all tissues.
