# Supplementary figures and images for: Human alpha defensin 5 is a candidate biomarker to delineate inflammatory bowel disease
Source: PLoS One. 2017 Aug 17;12(8):e0179710. doi: 10.1371/journal.pone.0179710 (PMC5560519; doi:10.1371/journal.pone.0179710)

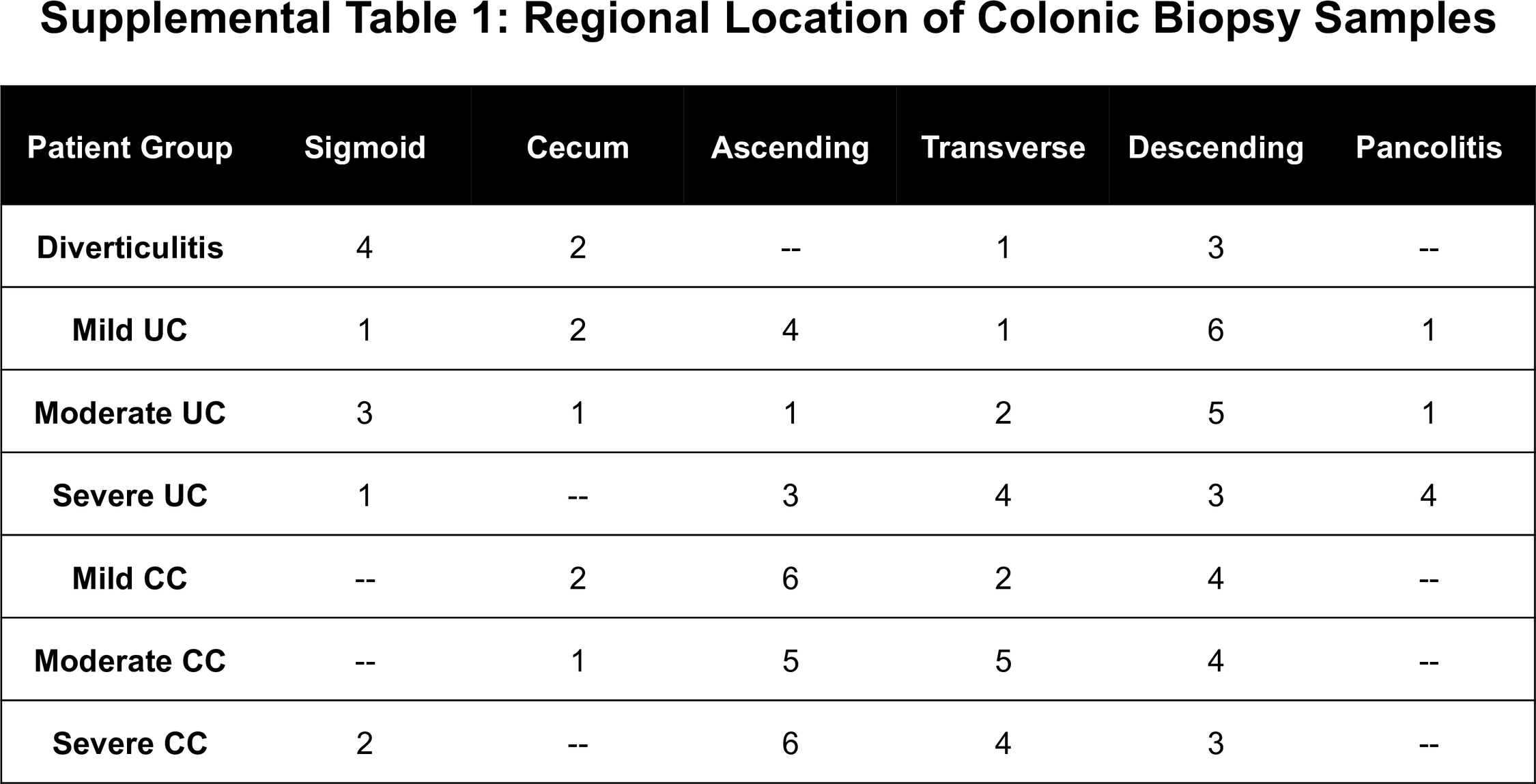

Supplement: S1 Table — Various full-thickness human biopsy samples were utilized to perform the experiments. This table shows the sample locations that were utilized in each patient group. (TIF) [file pone.0179710.s001.tif]

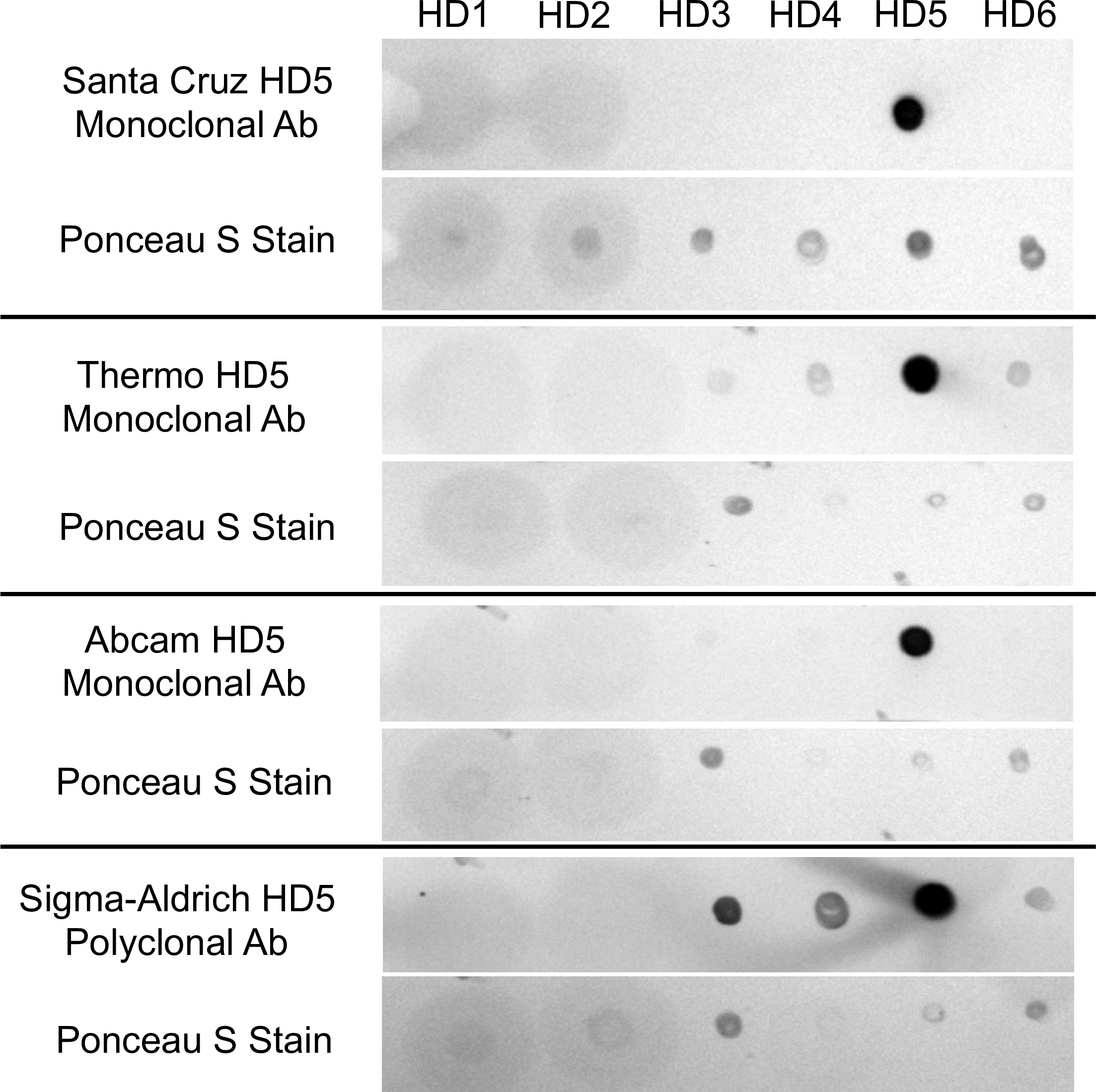

Supplement: S1 Fig — Dot blots were performed on recombinant HD1-6 with various commercial antibodies to determine specificity to HD5. Ponceau S Stain is used as a loading control. We found that the antibody from Santa Cruz was the most specific for HD5 of those tested. (TIF) [file pone.0179710.s003.tif]
