# Supplementary material for: Human alpha defensin 5 is a candidate biomarker to delineate inflammatory bowel disease
Source: PLoS One. 2017 Aug 17;12(8):e0179710. doi: 10.1371/journal.pone.0179710 (PMC5560519; doi:10.1371/journal.pone.0179710)
Supplement: S2 Table — A total of 484 genes were shown to be altered significantly +/- 2-fold between UC and CC according to the Affymetrix Microarray. All genes with p<0.05 are included in this table. (PDF) [file pone.0179710.s002.pdf]

Supplementary Table 1: Complete Table of Microaray Targets, in decending order of fold change difference

Gene Information	Gene Symbol	RefSeq	p-value (CC vs. UC)	Fold Change (CC vs. UC)
NM_021010 // DEFA5 // defensin, alpha 5, Paneth cell-specific // 8p23.1 // 1670	DEFA5	NM_021010	7.23E-05	31.0374
NM_002909 // REG1A // regenerating islet-derived 1 alpha // 2p12 // 5967 /// ENS	REG1A	NM_002909	0.00321456	21.9439
NM_138938 // REG3A // regenerating islet-derived 3 alpha // 2p12 // 5068 /// NM_	REG3A	NM_138938	0.000310891	17.3268
NM_001926 // DEFA6 // defensin, alpha 6, Paneth cell-specific // 8p23.1 // 1671	DEFA6	NM_001926	0.0024893	16.139
NM_058186 // FAM3B // family with sequence similarity 3, member B // 21q22.3 //	FAM3B	NM_058186	0.00116588	14.6887
NM_006507 // REG1B // regenerating islet-derived 1 beta // 2p12 // 5968 /// ENST	REG1B	NM_006507	0.0120953	13.9675
NM_001074 // UGT2B7 // UDP glucuronosyltransferase 2 family, polypeptide B7 // 4	UGT2B7	NM_001074	0.0154146	9.92532
NM_001285 // CLCA1 // chloride channel accessory 1 // 1p22.3 // 1179 /// ENST000	CLCA1	NM_001285	0.00297816	9.07579
NM_003122 // SPINK1 // serine peptidase inhibitor, Kazal type 1 // 5q32 // 6690	SPINK1	NM_003122	0.007176	7.60063
NM_001076 // UGT2B15 // UDP glucuronosyltransferase 2 family, polypeptide B15 //	UGT2B15	NM_001076	0.0169187	7.12294
NM_001076 // UGT2B15 // UDP glucuronosyltransferase 2 family, polypeptide B15 //	UGT2B15	NM_001076	0.0169187	7.12294
NM_000343 // SLC5A1 // solute carrier family 5 (sodium/glucose cotransporter), m	SLC5A1	NM_000343	0.00447091	7.0494
NM_000134 // FABP2 // fatty acid binding protein 2, intestinal // 4q28-q31 // 21	FABP2	NM_000134	0.0300574	6.63756
NM_000035 // ALDOB // aldolase B, fructose-bisphosphate // 9q21.3-q22.2 // 229 /	ALDOB	NM_000035	0.0444145	6.30502
NM_002770 // PRSS2 // protease, serine, 2 (trypsin 2) // 7q34 // 5645 /// ENST00	PRSS2	NM_002770	0.0052665	6.27999
NM_005379 // MYO1A // myosin IA // 12q13-q14 // 4640 /// ENST00000300119 // MYO1	MYO1A	NM_005379	0.00588172	5.72861
NM_007329 // DMBT1 // deleted in malignant brain tumors 1 // 10q26.13 // 1755 //	DMBT1	NM_007329	0.0365636	5.56609
NM_031457 // MS4A8B // membrane-spanning 4-domains, subfamily A, member 8B // 11	MS4A8B	NM_031457	0.00577952	5.34254
NM_001041 // SI // sucrase-isomaltase (alpha-glucosidase) // 3q25.2-q26.2 // 647	SI	NM_001041	0.0417578	5.23854
NM_000482 // APOA4 // apolipoprotein A-IV // 11q23 // 337 /// ENST00000357780 //	APOA4	NM_000482	0.0468523	5.15957
NM_006418 // OLFM4 // olfactomedin 4 // 13q14.3 // 10562 /// ENST00000219022 //	OLFM4	NM_006418	0.038931	5.05883
NM_000482 // APOA4 // apolipoprotein A-IV // 11q23 // 337 /// ENST00000357780 //	APOA4	NM_000482	0.0472178	4.92519
NM_004133 // HNF4G // hepatocyte nuclear factor 4, gamma // 8q21.11 // 3174 ///	HNF4G	NM_004133	0.0113549	4.8964
NM_017675 // CDHR2 // cadherin-related family member 2 // 5q35.2 // 54825 /// NM	CDHR2	NM_017675	0.00253568	4.82206
NM_005588 // MEP1A // meprin A, alpha (PABA peptide hydrolase) // 6p12-p11 // 42	MEP1A	NM_005588	0.0198087	4.78504
NM_002354 // EPCAM // epithelial cell adhesion molecule // 2p21 // 4072 /// ENST	EPCAM	NM_002354	0.0242383	4.77321
NM_001172312 // PLS1 // plastin 1 // 3q23 // 5357 /// NM_001145319 // PLS1 // pl	PLS1	NM_001172312	0.0155248	4.73894
NM_002354 // EPCAM // epithelial cell adhesion molecule // 2p21 // 4072 /// ENST	EPCAM	NM_002354	0.0297878	4.72533
NM_001150 // ANPEP // alanyl (membrane) aminopeptidase // 15q25-q26 // 290 /// E	ANPEP	NM_001150	0.0203087	4.58929
NM_001077 // UGT2B17 // UDP glucuronosyltransferase 2 family, polypeptide B17 //	UGT2B17	NM_001077	0.0267812	4.51157
NM_002591 // PCK1 // phosphoenolpyruvate carboxykinase 1 (soluble) // 20q13.31 /	PCK1	NM_002591	0.0333639	4.50793
NM_021804 // ACE2 // angiotensin I converting enzyme (peptidyl-dipeptidase A) 2	ACE2	NM_021804	0.0271919	4.49025
NM_024308 // DHRS11 // dehydrogenase/reductase (SDR family) member 11 // 17q12 /	DHRS11	NM_024308	0.0176773	4.41914
NM_019010 // KRT20 // keratin 20 // 17q21.2 // 54474 /// ENST00000167588 // KRT2	KRT20	NM_019010	0.026162	4.35459
ENST00000319509 // MUC3A // mucin 3A, cell surface associated // 7q22 // 4584 //	MUC3A	ENST00000319509	0.00353785	4.28484

Supplementary Table 1: Complete Table of Microaray Targets, in decending order of fold change difference

Gene Information	Gene Symbol	RefSeq	p-value (CC vs. UC)	Fold Change (CC vs. UC)
NM_000379 // XDH // xanthine dehydrogenase // 2p23.1 // 7498 /// ENST00000379416	XDH	NM_000379	0.00289109	4.17476
NM_007127 // VIL1 // villin 1 // 2q35 // 7429 /// ENST00000248444 // VIL1 // vil	VIL1	NM_007127	0.00825691	4.16925
NM_025130 // HKDC1 // hexokinase domain containing 1 // 10q22.1 // 80201 /// ENS	HKDC1	NM_025130	0.00344261	4.13874
NR_029578 // MIR192 // microRNA 192 // 11q13.1 // 406967	MIR192	NR_029578	0.00199884	4.12467
NM_004063 // CDH17 // cadherin 17, LI cadherin (liver-intestine) // 8q22.1 // 10	CDH17	NM_004063	0.0331015	4.12001
NM_024922 // CES3 // carboxylesterase 3 // 16q22.1 // 23491 /// NM_001185177 //	CES3	NM_024922	0.0022354	4.11886
NM_033049 // MUC13 // mucin 13, cell surface associated // 3q21.2 // 56667 /// E	MUC13	NM_033049	0.0271079	4.11287
NM_000888 // ITGB6 // integrin, beta 6 // 2q24.2 // 3694 /// ENST00000283249 //	ITGB6	NM_000888	0.000602949	4.09738
NM_004963 // GUCY2C // guanylate cyclase 2C (heat stable enterotoxin receptor) /	GUCY2C	NM_004963	0.00645462	4.0793
NM_004293 // GDA // guanine deaminase // 9q21.13 // 9615 /// ENST00000358399 //	GDA	NM_004293	0.0208862	4.0739
NM_001307 // CLDN7 // claudin 7 // 17p13 // 1366 /// NM_001185022 // CLDN7 // cl	CLDN7	NM_001307	0.0213404	4.06183
NR_033807 // CYP3A5 // cytochrome P450, family 3, subfamily A, polypeptide 5 //	CYP3A5	NR_033807	0.0046334	4.04376
NM_021924 // CDHR5 // cadherin-related family member 5 // 11p15.5 // 53841 /// N	CDHR5	NM_021924	0.00480695	3.97925
NM_001010922 // BCL2L15 // BCL2-like 15 // 1p13.2 // 440603 /// ENST00000393316	BCL2L15	NM_001010922	0.027053	3.96946
NM_020770 // CGN // cingulin // 1q21 // 57530 /// ENST00000271636 // CGN // cing	CGN	NM_020770	0.00129584	3.94184
NM_032787 // GPR128 // G protein-coupled receptor 128 // 3q12.2 // 84873 /// ENS	GPR128	NM_032787	0.00779494	3.93937
NM_138933 // A1CF // APOBEC1 complementation factor // 10q11.23 // 29974 /// NM_	A1CF	NM_138933	0.00976589	3.79699
NM_152311 // CLRN3 // clarin 3 // 10q26.2 // 119467 /// ENST00000368671 // CLRN3	CLRN3	NM_152311	0.0132404	3.74982
NM_007072 // HHLA2 // HERV-H LTR-associating 2 // 3q13.13 // 11148 /// ENST00000	HHLA2	NM_007072	0.0139075	3.74668
NM_003399 // XPNPEP2 // X-prolyl aminopeptidase (aminopeptidase P) 2, membrane-b	XPNPEP2	NM_003399	0.0359348	3.73179
NM_021258 // IL22RA1 // interleukin 22 receptor, alpha 1 // 1p36.11 // 58985 ///	IL22RA1	NM_021258	0.00520995	3.72759
NM_000149 // FUT3 // fucosyltransferase 3 (galactoside 3(4)-L-fucosyltransferase	FUT3	NM_000149	0.0106419	3.70158
NM_002644 // PIGR // polymeric immunoglobulin receptor // 1q31-q41 // 5284 /// E	PIGR	NM_002644	0.0363588	3.68869
NM_001136503 // C19orf77 // chromosome 19 open reading frame 77 // 19p13.3 // 28	C19orf77	NM_001136503	0.0114867	3.6586
NR_024626 // C17orf73 // chromosome 17 open reading frame 73 // 17q21.33 // 5501	C17orf73	NR_024626	0.00240775	3.64138
NM_020973 // GBA3 // glucosidase, beta, acid 3 (cytosolic) // 4p15.2 // 57733 //	GBA3	NM_020973	0.0362758	3.63402
NM_023944 // CYP4F12 // cytochrome P450, family 4, subfamily F, polypeptide 12 /	CYP4F12	NM_023944	0.00468827	3.62246
NM_024320 // PRR15L // proline rich 15-like // 17q21.32 // 79170 /// ENST0000030	PRR15L	NM_024320	0.0331566	3.60367
NM_005495 // SLC17A4 // solute carrier family 17 (sodium phosphate), member 4 //	SLC17A4	NM_005495	0.0299201	3.59753
NM_001135099 // TMPRSS2 // transmembrane protease, serine 2 // 21q22.3 // 7113 /	TMPRSS2	NM_001135099	0.0351257	3.57585
NM_001193434 // C10orf81 // chromosome 10 open reading frame 81 // 10q25.3 // 79	C10orf81	NM_001193434	0.00228381	3.5687
NM_001935 // DPP4 // dipeptidyl-peptidase 4 // 2q24.3 // 1803 /// ENST0000036053	DPP4	NM_001935	0.0302652	3.49144
NM_001644 // APOBEC1 // apolipoprotein B mRNA editing enzyme, catalytic polypept	APOBEC1	NM_001644	0.0138008	3.48792
NM_004360 // CDH1 // cadherin 1, type 1, E-cadherin (epithelial) // 16q22.1 // 9	CDH1	NM_004360	0.010781	3.48059
NM_024921 // POF1B // premature ovarian failure, 1B // Xq21.2 // 79983 /// ENST0	POF1B	NM_024921	0.0313161	3.44457

Supplementary Table 1: Complete Table of Microaray Targets, in decending order of fold change difference

Gene Information	Gene Symbol	RefSeq	p-value (CC vs. UC)	Fold Change (CC vs. UC)
NM_002416 // CXCL9 // chemokine (C-X-C motif) ligand 9 // 4q21 // 4283 /// ENST0	CXCL9	NM_002416	0.00248734	3.44146
NM_014479 // ADAMDEC1 // ADAM-like, decysin 1 // 8p21.2 // 27299 /// NM_00114527	ADAMDEC1	NM_014479	0.00203661	3.42469
NM_001112706 // SCIN // scinderin // 7p21.3 // 85477 /// NM_033128 // SCIN // sc	SCIN	NM_001112706	0.00493508	3.3952
NR_024345 // NCRNA00262 // non-protein coding RNA 262 // 12q24.31 // 283460	NCRNA00262	NR_024345	0.037473	3.39502
NM_002273 // KRT8 // keratin 8 // 12q13 // 3856 /// ENST00000293308 // KRT8 // k	KRT8	NM_002273	0.0146545	3.39222
NM_001038603 // MARVELD2 // MARVEL domain containing 2 // 5q13.2 // 153562 /// E	MARVELD2	NM_001038603	0.0179974	3.37682
NM_001038603 // MARVELD2 // MARVEL domain containing 2 // 5q13.2 // 153562 /// E	MARVELD2	NM_001038603	0.0179974	3.37682
NM_144575 // CAPN13 // calpain 13 // 2p22-p21 // 92291 /// ENST00000295055 // CA	CAPN13	NM_144575	0.013239	3.36885
NM_022129 // PBLD // phenazine biosynthesis-like protein domain containing // 10	PBLD	NM_022129	0.00497915	3.3666
NM_000775 // CYP2J2 // cytochrome P450, family 2, subfamily J, polypeptide 2 //	CYP2J2	NM_000775	0.0196093	3.36302
NM_001135195 // SLC39A5 // solute carrier family 39 (metal ion transporter), mem	SLC39A5	NM_001135195	0.00623473	3.34227
NM_138788 // TMEM45B // transmembrane protein 45B // 11q24.3 // 120224 /// ENST0	TMEM45B	NM_138788	0.0306305	3.33725
NM_176813 // AGR3 // anterior gradient homolog 3 (Xenopus laevis) // 7p21.1 // 1	AGR3	NM_176813	0.0400823	3.32266
NM_022901 // LRRC19 // leucine rich repeat containing 19 // 9p21.2 // 64922 ///	LRRC19	NM_022901	0.0294679	3.31296
NM_139053 // EPS8L3 // EPS8-like 3 // 1p13.3 // 79574 /// NM_133181 // EPS8L3 //	EPS8L3	NM_139053	0.00371579	3.29224
NM_017697 // ESRP1 // epithelial splicing regulatory protein 1 // 8q22.1 // 5484	ESRP1	NM_017697	0.0234665	3.27492
NM_002457 // MUC2 // mucin 2, oligomeric mucus/gel-forming // 11p15.5 // 4583 //	MUC2	NM_002457	0.0182535	3.26416
NR_001296 // TRY6 // trypsinogen C // 7q34 // 154754 /// NM_002770 // PRSS2 // p	TRY6	NR_001296	0.0203767	3.24356
NM_002773 // PRSS8 // protease, serine, 8 // 16p11.2 // 5652 /// ENST00000317508	PRSS8	NM_002773	0.0131026	3.2405
NM_025214 // CCDC68 // coiled-coil domain containing 68 // 18q21 // 80323 /// NM	CCDC68	NM_025214	0.00627753	3.2264
NM_001943 // DSG2 // desmoglein 2 // 18q12.1 // 1829 /// ENST00000261590 // DSG2	DSG2	NM_001943	0.0357587	3.22627
NM_000772 // CYP2C18 // cytochrome P450, family 2, subfamily C, polypeptide 18 /	CYP2C18	NM_000772	0.0100284	3.20876
NM_000767 // CYP2B6 // cytochrome P450, family 2, subfamily B, polypeptide 6 //	CYP2B6	NM_000767	0.00589423	3.19484
NM_016234 // ACSL5 // acyl-CoA synthetase long-chain family member 5 // 10q25.1-	ACSL5	NM_016234	0.00353915	3.19242
NM_145865 // ANKS4B // ankyrin repeat and sterile alpha motif domain containing	ANKS4B	NM_145865	0.027168	3.16823
NM_032579 // RETNLB // resistin like beta // 3q13.1 // 84666 /// ENST00000295755	RETNLB	NM_032579	0.0226491	3.14305
NM_021978 // ST14 // suppression of tumorigenicity 14 (colon carcinoma) // 11q24	ST14	NM_021978	0.0143682	3.14171
NM_000492 // CFTR // cystic fibrosis transmembrane conductance regulator (ATP-bi	CFTR	NM_000492	0.0330127	3.13524
NM_018842 // BAIAP2L1 // BAI1-associated protein 2-like 1 // 7q22.1 // 55971 ///	BAIAP2L1	NM_018842	0.00626097	3.13099
NM_001165958 // GSDMB // gasdermin B // 17q12 // 55876 /// NM_001042471 // GSDMB	GSDMB	NM_001165958	0.0013942	3.1309
NM_024422 // DSC2 // desmocollin 2 // 18q12.1 // 1824 /// NM_004949 // DSC2 // d	DSC2	NM_024422	0.0115939	3.11862
NM_006017 // PROM1 // prominin 1 // 4p15.32 // 8842 /// NM_001145847 // PROM1 //	PROM1	NM_006017	0.0116042	3.10273
NM_017878 // HRASLS2 // HRAS-like suppressor 2 // 11q12.3 // 54979 /// ENST00000	HRASLS2	NM_017878	0.0267887	3.09847
NM_002203 // ITGA2 // integrin, alpha 2 (CD49B, alpha 2 subunit of VLA-2 recepto	ITGA2	NM_002203	0.00793505	3.07141
NM_005123 // NR1H4 // nuclear receptor subfamily 1, group H, member 4 // 12q23.1	NR1H4	NM_005123	0.0456782	3.06865

Supplementary Table 1: Complete Table of Microaray Targets, in decending order of fold change difference

Gene Information	Gene Symbol	RefSeq	p-value (CC vs. UC)	Fold Change (CC vs. UC)
NM_001145862 // MTMR11 // myotubularin related protein 11 // 1q12-q21 // 10903 /	MTMR11	NM_001145862	0.00116554	3.03455
NM_018414 // ST6GALNAC1 // ST6 (alpha-N-acetyl-neuraminyl-2,3-beta-galactosyl-1,	ST6GALNAC1	NM_018414	0.0240185	3.0202
NM_001080527 // MYO7B // myosin VIIB // 2q21.1 // 4648 /// ENST00000428314 // MY	MYO7B	NM_001080527	0.00130692	2.99927
NM_002153 // HSD17B2 // hydroxysteroid (17-beta) dehydrogenase 2 // 16q24.1-q24.	HSD17B2	NM_002153	0.0213389	2.99803
AK095678 // LOC151009 // hypothetical LOC151009 // 2q13 // 151009 /// AK056084 /	LOC151009	AK095678	0.000466288	2.99502
NM_000769 // CYP2C19 // cytochrome P450, family 2, subfamily C, polypeptide 19 /	CYP2C19	NM_000769	0.0193957	2.99186
NM_000790 // DDC // dopa decarboxylase (aromatic L-amino acid decarboxylase) //	DDC	NM_000790	0.0257511	2.98778
NM_001143948 // C6orf105 // chromosome 6 open reading frame 105 // 6p24.1 // 848	C6orf105	NM_001143948	0.0220945	2.95786
NM_001015001 // CKMT1A // creatine kinase, mitochondrial 1A // 15q15 // 548596 /	CKMT1A	NM_001015001	0.042629	2.95709
NM_001015001 // CKMT1A // creatine kinase, mitochondrial 1A // 15q15 // 548596 /	CKMT1A	NM_001015001	0.042629	2.95709
NM_019893 // ASAH2 // N-acylsphingosine amidohydrolase (non-lysosomal ceramidase	ASAH2	NM_019893	0.0167497	2.95643
NM_001002236 // SERPINA1 // serpin peptidase inhibitor, clade A (alpha-1 antipro	SERPINA1	NM_001002236	0.0170929	2.94245
NM_002031 // FRK // fyn-related kinase // 6q21-q22.3 // 2444 /// ENST00000368626	FRK	NM_002031	0.0177896	2.93608
NM_001190482 // PCSK5 // proprotein convertase subtilisin/kexin type 5 // 9q21.3	PCSK5	NM_001190482	0.00160967	2.92603
NM_004415 // DSP // desmoplakin // 6p24 // 1832 /// NM_001008844 // DSP // desmo	DSP	NM_004415	0.0116502	2.91732
NM_004591 // CCL20 // chemokine (C-C motif) ligand 20 // 2q33-q37 // 6364 /// NM	CCL20	NM_004591	0.0229351	2.91511
NM_000561 // GSTM1 // glutathione S-transferase mu 1 // 1p13.3 // 2944 /// NM_14	GSTM1	NM_000561	0.032505	2.91233
NM_000927 // ABCB1 // ATP-binding cassette, sub-family B (MDR/TAP), member 1 //	ABCB1	NM_000927	0.03279	2.89709
NM_000187 // HGD // homogentisate 1,2-dioxygenase // 3q13.33 // 3081 /// ENST000	HGD	NM_000187	0.0180393	2.8961
NM_000187 // HGD // homogentisate 1,2-dioxygenase // 3q13.33 // 3081 /// ENST000	HGD	NM_000187	0.0180393	2.8961
NM_153676 // USH1C // Usher syndrome 1C (autosomal recessive, severe) // 11p14.3	USH1C	NM_153676	0.00547469	2.88241
NM_005624 // CCL25 // chemokine (C-C motif) ligand 25 // 19p13.2 // 6370 /// ENS	CCL25	NM_005624	0.0492359	2.86049
NM_004174 // SLC9A3 // solute carrier family 9 (sodium/hydrogen exchanger), memb	SLC9A3	NM_004174	0.0173616	2.8567
NM_001306 // CLDN3 // claudin 3 // 7q11.23 // 1365 /// ENST00000395145 // CLDN3	CLDN3	NM_001306	0.0490185	2.84657
NM_001114309 // ELF3 // E74-like factor 3 (ets domain transcription factor, epit	ELF3	NM_001114309	0.00265363	2.84098
NM_000507 // FBP1 // fructose-1,6-bisphosphatase 1 // 9q22.3 // 2203 /// NM_0011	FBP1	NM_000507	0.022351	2.83767
NM_025257 // SLC44A4 // solute carrier family 44, member 4 // 6p21.3 // 80736 //	SLC44A4	NM_025257	0.0415598	2.83697
NM_025257 // SLC44A4 // solute carrier family 44, member 4 // 6p21.3 // 80736 //	SLC44A4	NM_025257	0.0415598	2.83697
NM_025257 // SLC44A4 // solute carrier family 44, member 4 // 6p21.3 // 80736 //	SLC44A4	NM_025257	0.0415598	2.83697
NM_001017970 // TMEM30B // transmembrane protein 30B // 14q23.1 // 161291 /// EN	TMEM30B	NM_001017970	0.00717685	2.83259
NM_003963 // TM4SF5 // transmembrane 4 L six family member 5 // 17p13.3 // 9032	TM4SF5	NM_003963	0.0295851	2.82875
NM_002242 // KCNJ13 // potassium inwardly-rectifying channel, subfamily J, membe	KCNJ13	NM_002242	0.0400838	2.82471
NM_017655 // GIPC2 // GIPC PDZ domain containing family, member 2 // 1p31.1 // 5	GIPC2	NM_017655	0.0155498	2.81938
NM_001127605 // LIPA // lipase A, lysosomal acid, cholesterol esterase // 10q23.	LIPA	NM_001127605	0.000449938	2.81611
NM_001249 // ENTPD5 // ectonucleoside triphosphate diphosphohydrolase 5 // 14q24	ENTPD5	NM_001249	0.0118697	2.81265

Supplementary Table 1: Complete Table of Microaray Targets, in decending order of fold change difference

Gene Information	Gene Symbol	RefSeq	p-value (CC vs. UC)	Fold Change (CC vs. UC)
NM_005358 // LMO7 // LIM domain 7 // 13q22.2 // 4008 /// NM_015842 // LMO7 // LI	LMO7	NM_005358	0.00460576	2.80795
NM_018667 // SMPD3 // sphingomyelin phosphodiesterase 3, neutral membrane (neutr	SMPD3	NM_018667	0.00228114	2.80665
NM_004563 // PCK2 // phosphoenolpyruvate carboxykinase 2 (mitochondrial) // 14q1	PCK2	NM_004563	0.00983672	2.79262
NM_003657 // BCAS1 // breast carcinoma amplified sequence 1 // 20q13.2 // 8537 /	BCAS1	NM_003657	0.0213345	2.78368
NM_024850 // BTNL8 // butyrophilin-like 8 // 5q35.3 // 79908 /// NM_001040462 //	BTNL8	NM_024850	0.0446038	2.7769
NM_020672 // S100A14 // S100 calcium binding protein A14 // 1q21.3 // 57402 ///	S100A14	NM_020672	0.0202797	2.77156
NM_033229 // TRIM15 // tripartite motif-containing 15 // 6p21.3 // 89870 /// ENS	TRIM15	NM_033229	0.0097609	2.77095
NM_033229 // TRIM15 // tripartite motif-containing 15 // 6p21.3 // 89870 /// ENS	TRIM15	NM_033229	0.0097609	2.77095
NM_033229 // TRIM15 // tripartite motif-containing 15 // 6p21.3 // 89870 /// ENS	TRIM15	NM_033229	0.0097609	2.77095
NM_001144060 // NHSL1 // NHS-like 1 // 6q23.3 // 57224 /// NM_020464 // NHSL1 //	NHSL1	NM_001144060	0.0124428	2.7705
NM_003869 // CES2 // carboxylesterase 2 // 16q22.1 // 8824 /// NR_036684 // CES2	CES2	NM_003869	0.0197746	2.76326
NM_199187 // KRT18 // keratin 18 // 12q13 // 3875 /// NM_000224 // KRT18 // kera	KRT18	NM_199187	0.0272938	2.7567
NM_002842 // PTPRH // protein tyrosine phosphatase, receptor type, H // 19q13.4	PTPRH	NM_002842	0.00126103	2.75623
NM_001105248 // TMC5 // transmembrane channel-like 5 // 16p12.3 // 79838 /// NM_	TMC5	NM_001105248	0.015439	2.74553
NM_001145809 // MYH14 // myosin, heavy chain 14, non-muscle // 19q13.33 // 79784	MYH14	NM_001145809	0.00203315	2.74198
NM_001054 // SULT1A2 // sulfotransferase family, cytosolic, 1A, phenol-preferrin	SULT1A2	NM_001054	0.0273843	2.73
NM_024850 // BTNL8 // butyrophilin-like 8 // 5q35.3 // 79908 /// NM_001159708 //	BTNL8	NM_024850	0.0433332	2.7165
NM_006147 // IRF6 // interferon regulatory factor 6 // 1q32.3-q41 // 3664 /// EN	IRF6	NM_006147	0.00663477	2.71435
NM_000457 // HNF4A // hepatocyte nuclear factor 4, alpha // 20q13.12 // 3172 ///	HNF4A	NM_000457	0.00414138	2.70616
NM_138809 // CMBL // carboxymethylenebutenolidase homolog (Pseudomonas) // 5p15.	CMBL	NM_138809	0.0336993	2.69623
NM_001080467 // MYO5B // myosin VB // 18q21 // 4645 /// ENST00000285039 // MYO5B	MYO5B	NM_001080467	0.00639465	2.69568
NM_153274 // BEST4 // bestrophin 4 // 1p33-p32.3 // 266675 /// ENST00000372207 /	BEST4	NM_153274	0.0313639	2.68747
NM_020775 // KIAA1324 // KIAA1324 // 1p13.3 // 57535 /// ENST00000234923 // KIAA	KIAA1324	NM_020775	0.0214297	2.68133
NM_001004320 // TMEM195 // transmembrane protein 195 // 7p21.2 // 392636 /// ENS	TMEM195	NM_001004320	0.0149666	2.67293
NM_001091 // ABP1 // amiloride binding protein 1 (amine oxidase (copper-containi	ABP1	NM_001091	0.0487109	2.66772
NM_016245 // HSD17B11 // hydroxysteroid (17-beta) dehydrogenase 11 // 4q22.1 //	HSD17B11	NM_016245	0.0216559	2.66473
NM_006144 // GZMA // granzyme A (granzyme 1, cytotoxic T-lymphocyte-associated s	GZMA	NM_006144	0.00618242	2.66284
NM_001039372 // HEPACAM2 // HEPACAM family member 2 // 7q21.3 // 253012 /// NM_1	HEPACAM2	NM_001039372	0.0201907	2.6524
NM_001197097 // PRSS3 // protease, serine, 3 // 9p11.2 // 5646 /// NM_007343 //	PRSS3	NM_001197097	0.0173103	2.63924
NM_012214 // MGAT4A // mannosyl (alpha-1,3-)-glycoprotein beta-1,4-N-acetylgluco	MGAT4A	NM_012214	0.00113208	2.62742
NM_019894 // TMPRSS4 // transmembrane protease, serine 4 // 11q23.3 // 56649 ///	TMPRSS4	NM_019894	0.0362683	2.60764
NM_003810 // TNFSF10 // tumor necrosis factor (ligand) superfamily, member 10 //	TNFSF10	NM_003810	0.0129809	2.60509
NM_022842 // CDCP1 // CUB domain containing protein 1 // 3p21.31 // 64866 /// NM	CDCP1	NM_022842	0.0167874	2.60268
NM_001136493 // MFSD2A // major facilitator superfamily domain containing 2A //	MFSD2A	NM_001136493	0.00343618	2.59815
NM_018265 // C1orf106 // chromosome 1 open reading frame 106 // 1q32.1 // 55765	C1orf106	NM_018265	0.00613223	2.59677

Supplementary Table 1: Complete Table of Microaray Targets, in decending order of fold change difference

Gene Information	Gene Symbol	RefSeq	p-value (CC vs. UC)	Fold Change (CC vs. UC)
NM_000063 // C2 // complement component 2 // 6p21.3 // 717 /// NM_001145903 // C	C2	NM_000063	0.0117239	2.59406
NM_000063 // C2 // complement component 2 // 6p21.3 // 717 /// NM_001145903 // C	C2	NM_000063	0.0117239	2.59406
NM_000625 // NOS2 // nitric oxide synthase 2, inducible // 17q11.2-q12 // 4843 /	NOS2	NM_000625	0.0089305	2.59304
NM_001677 // ATP1B1 // ATPase, Na+/K+ transporting, beta 1 polypeptide // 1q24 /	ATP1B1	NM_001677	0.0131783	2.58871
NM_004751 // GCNT3 // glucosaminyl (N-acetyl) transferase 3, mucin type // 15q21	GCNT3	NM_004751	0.0432197	2.58761
NM_002021 // FMO1 // flavin containing monooxygenase 1 // 1q24.3 // 2326 /// ENS	FMO1	NM_002021	0.0408097	2.57646
NM_033292 // CASP1 // caspase 1, apoptosis-related cysteine peptidase (interleuk	CASP1	NM_033292	0.00634065	2.57013
NM_147161 // ACOT11 // acyl-CoA thioesterase 11 // 1p32.3 // 26027 /// ENST00000	ACOT11	NM_147161	0.0462671	2.53682
NM_001039112 // FER1L6 // fer-1-like 6 (C. elegans) // 8q24.1 // 654463 /// ENST	FER1L6	NM_001039112	0.0413201	2.53444
NM_212543 // B4GALT4 // UDP-Gal:betaGlcNAc beta 1,4- galactosyltransferase, poly	B4GALT4	NM_212543	0.00083206	2.53146
NM_182762 // MACC1 // metastasis associated in colon cancer 1 // 7p21.1 // 34638	MACC1	NM_182762	0.0113734	2.52994
NM_001461 // FMO5 // flavin containing monooxygenase 5 // 1q21.1 // 2330 /// NM_	FMO5	NM_001461	0.0227505	2.52925
NM_031219 // HDHD3 // haloacid dehalogenase-like hydrolase domain containing 3 /	HDHD3	NM_031219	0.00048055	2.52696
NM_001010872 // FAM83B // family with sequence similarity 83, member B // 6p12.1	FAM83B	NM_001010872	0.00806204	2.52496
NM_024533 // CHST5 // carbohydrate (N-acetylglucosamine 6-O) sulfotransferase 5	CHST5	NM_024533	0.026327	2.51739
NM_000063 // C2 // complement component 2 // 6p21.3 // 717 /// NM_001145903 // C	C2	NM_000063	0.0114041	2.51419
NM_004624 // VIPR1 // vasoactive intestinal peptide receptor 1 // 3p22 // 7433 /	VIPR1	NM_004624	0.00331244	2.50863
NM_004572 // PKP2 // plakophilin 2 // 12p11 // 5318 /// NM_001005242 // PKP2 //	PKP2	NM_004572	0.042448	2.49612
NM_032521 // PARD6B // par-6 partitioning defective 6 homolog beta (C. elegans)	PARD6B	NM_032521	0.00395798	2.49598
NM_024915 // GRHL2 // grainyhead-like 2 (Drosophila) // 8q22.3 // 79977 /// ENST	GRHL2	NM_024915	0.00624177	2.49455
NM_003982 // SLC7A7 // solute carrier family 7 (cationic amino acid transporter,	SLC7A7	NM_003982	0.00813405	2.49274
NM_198584 // CA13 // carbonic anhydrase XIII // 8q21.2 // 377677 /// ENST0000032	CA13	NM_198584	0.00510852	2.48988
ENST00000319509 // MUC3A // mucin 3A, cell surface associated // 7q22 // 4584 //	MUC3A	ENST00000319509	0.0135883	2.4817
NM_021102 // SPINT2 // serine peptidase inhibitor, Kunitz type, 2 // 19q13.1 //	SPINT2	NM_021102	0.0219176	2.48131
NM_080489 // SDCBP2 // syndecan binding protein (syntenin) 2 // 20p13 // 27111 /	SDCBP2	NM_080489	0.000789754	2.47862
NM_001144967 // NEDD4L // neural precursor cell expressed, developmentally down-	NEDD4L	NM_001144967	0.0227827	2.47791
NM_001982 // ERBB3 // v-erb-b2 erythroblastic leukemia viral oncogene homolog 3	ERBB3	NM_001982	0.0175723	2.47531
NM_000240 // MAOA // monoamine oxidase A // Xp11.3 // 4128 /// ENST00000338702 /	MAOA	NM_000240	0.0446884	2.47082
NM_182960 // PRELID2 // PRELI domain containing 2 // 5q32 // 153768 /// NM_13849	PRELID2	NM_182960	0.00837834	2.47032
NM_017720 // STAP2 // signal transducing adaptor family member 2 // 19p13.3 // 5	STAP2	NM_017720	0.016285	2.46781
NM_138700 // TRIM40 // tripartite motif-containing 40 // 6p22.1 // 135644 /// EN	TRIM40	NM_138700	0.0336507	2.45989
NM_000050 // ASS1 // argininosuccinate synthase 1 // 9q34.1 // 445 /// NM_054012	ASS1	NM_000050	0.0132614	2.43678
NM_005021 // ENPP3 // ectonucleotide pyrophosphatase/phosphodiesterase 3 // 6q22	ENPP3	NM_005021	0.0149678	2.43651
NM_001130080 // IFI27 // interferon, alpha-inducible protein 27 // 14q32 // 3429	IFI27	NM_001130080	0.0140236	2.43613
NM_001979 // EPHX2 // epoxide hydrolase 2, cytoplasmic // 8p21 // 2053 /// BC011	EPHX2	NM_001979	0.00690804	2.43531

Supplementary Table 1: Complete Table of Microaray Targets, in decending order of fold change difference

Gene Information	Gene Symbol	RefSeq	p-value (CC vs. UC)	Fold Change (CC vs. UC)
NM_017700 // ARHGEF38 // Rho guanine nucleotide exchange factor (GEF) 38 // 4q24	ARHGEF38	NM_017700	0.00476968	2.42966
NM_019080 // NDFIP2 // Nedd4 family interacting protein 2 // 13q31.1 // 54602 //	NDFIP2	NM_019080	0.00576011	2.42832
NM_001135181 // SLC5A9 // solute carrier family 5 (sodium/glucose cotransporter)	SLC5A9	NM_001135181	0.0296431	2.42215
NM_032717 // AGPAT9 // 1-acylglycerol-3-phosphate O-acyltransferase 9 // 4q21.23	AGPAT9	NM_032717	0.0147877	2.41843
NM_001145303 // TMC4 // transmembrane channel-like 4 // 19q13.42 // 147798 /// N	TMC4	NM_001145303	0.00110774	2.41442
NM_138700 // TRIM40 // tripartite motif-containing 40 // 6p22.1 // 135644 /// EN	TRIM40	NM_138700	0.0250665	2.41358
NM_138700 // TRIM40 // tripartite motif-containing 40 // 6p22.1 // 135644 /// EN	TRIM40	NM_138700	0.0250665	2.41358
NM_203463 // LASS6 // LAG1 homolog, ceramide synthase 6 // 2q24.3 // 253782 ///	LASS6	NM_203463	0.00156196	2.41203
NM_001730 // KLF5 // Kruppel-like factor 5 (intestinal) // 13q22.1 // 688 /// EN	KLF5	NM_001730	0.0129015	2.40278
NM_001265 // CDX2 // caudal type homeobox 2 // 13q12.3 // 1045 /// ENST000003810	CDX2	NM_001265	0.0471437	2.402
NM_000239 // LYZ // lysozyme // 12q15 // 4069 /// ENST00000261267 // LYZ // lyso	LYZ	NM_000239	0.0118582	2.39899
NM_022772 // EPS8L2 // EPS8-like 2 // 11p15.5 // 64787 /// ENST00000318562 // EP	EPS8L2	NM_022772	0.00191717	2.39231
NM_025153 // ATP10B // ATPase, class V, type 10B // 5q34 // 23120 /// ENST000003	ATP10B	NM_025153	0.0273664	2.38677
NM_178445 // CCRL1 // chemokine (C-C motif) receptor-like 1 // 3q22 // 51554 ///	CCRL1	NM_178445	0.0328488	2.38032
NM_001031803 // LLGL2 // lethal giant larvae homolog 2 (Drosophila) // 17q25.1 /	LLGL2	NM_001031803	0.00351395	2.36948
NM_175058 // PLEKHA7 // pleckstrin homology domain containing, family A member 7	PLEKHA7	NM_175058	0.00170237	2.36502
NM_006714 // SMPDL3A // sphingomyelin phosphodiesterase, acid-like 3A // 6q22.31	SMPDL3A	NM_006714	0.0236138	2.36218
NR_024158 // LOC25845 // hypothetical LOC25845 // 5p15.33 // 25845 /// ENST00000	LOC25845	NR_024158	0.0297858	2.35341
NM_016339 // RAPGEFL1 // Rap guanine nucleotide exchange factor (GEF)-like 1 //	RAPGEFL1	NM_016339	0.026897	2.3526
NM_015888 // HOOK1 // hook homolog 1 (Drosophila) // 1p32.1 // 51361 /// ENST000	HOOK1	NM_015888	0.0336071	2.34842
NM_138737 // HEPH // hephaestin // Xq11-q12 // 9843 /// NM_001130860 // HEPH //	HEPH	NM_138737	0.0118198	2.34595
NM_012079 // DGAT1 // diacylglycerol O-acyltransferase 1 // 8q24.3 // 8694 /// E	DGAT1	NM_012079	0.023252	2.34522
NM_012079 // DGAT1 // diacylglycerol O-acyltransferase 1 // 8q24.3 // 8694 /// E	DGAT1	NM_012079	0.023252	2.34522
NM_001017535 // VDR // vitamin D (1,25- dihydroxyvitamin D3) receptor // 12q13.1	VDR	NM_001017535	0.0115491	2.34153
NM_001029874 // REP15 // RAB15 effector protein // 12p11.22 // 387849 /// ENST00	REP15	NM_001029874	0.0477963	2.33656
NM_198495 // CTAGE4 // CTAGE family, member 4 // 7q35 // 100128553 /// NM_001145	CTAGE4	NM_198495	0.00065154	2.33596
NM_006548 // IGF2BP2 // insulin-like growth factor 2 mRNA binding protein 2 // 3	IGF2BP2	NM_006548	8.80E-05	2.33476
NM_002985 // CCL5 // chemokine (C-C motif) ligand 5 // 17q11.2-q12 // 6352 /// E	CCL5	NM_002985	0.0247261	2.33002
NM_001005328 // OR2A7 // olfactory receptor, family 2, subfamily A, member 7 //	OR2A7	NM_001005328	0.00337105	2.32021
NM_018284 // GBP3 // guanylate binding protein 3 // 1p22.2 // 2635 /// ENST00000	GBP3	NM_018284	0.013933	2.31798
NM_002829 // PTPN3 // protein tyrosine phosphatase, non-receptor type 3 // 9q31	PTPN3	NM_002829	0.0212048	2.31511
NM_021073 // BMP5 // bone morphogenetic protein 5 // 6p12.1 // 653 /// ENST00000	BMP5	NM_021073	0.0201876	2.31001
NM_178176 // MOGAT3 // monoacylglycerol O-acyltransferase 3 // 7q22.1 // 346606	MOGAT3	NM_178176	0.00641018	2.30988
NM_000666 // ACY1 // aminoacylase 1 // 3p21.1 // 95 /// L07548 // ACY1 // aminoa	ACY1	NM_000666	0.0261486	2.30581
NM_001098634 // RBM47 // RNA binding motif protein 47 // 4p14 // 54502 /// NM_01	RBM47	NM_001098634	0.00857247	2.30203

Supplementary Table 1: Complete Table of Microaray Targets, in decending order of fold change difference

Gene Information	Gene Symbol	RefSeq	p-value (CC vs. UC)	Fold Change (CC vs. UC)
NM_080658 // ACY3 // aspartoacylase (aminocyclase) 3 // 11q13.2 // 91703 /// ENS	ACY3	NM_080658	0.0498753	2.301
NR_003587 // MYO15B // myosin XVB pseudogene // 17q25.1 // 80022 /// BC027875 //	MYO15B	NR_003587	0.00759021	2.29754
NM_005435 // ARHGEF5 // Rho guanine nucleotide exchange factor (GEF) 5 // 7q33-q	ARHGEF5	NM_005435	0.00766916	2.29684
NM_005435 // ARHGEF5 // Rho guanine nucleotide exchange factor (GEF) 5 // 7q33-q	ARHGEF5	NM_005435	0.00846455	2.29311
NM_001017967 // MARVELD3 // MARVEL domain containing 3 // 16q22.2 // 91862 /// N	MARVELD3	NM_001017967	0.0124186	2.2921
NM_003389 // CORO2A // coronin, actin binding protein, 2A // 9q22.3 // 7464 ///	CORO2A	NM_003389	0.0203606	2.28709
NM_031469 // SH3BGRL2 // SH3 domain binding glutamic acid-rich protein like 2 //	SH3BGRL2	NM_031469	0.0214373	2.27245
NM_030766 // BCL2L14 // BCL2-like 14 (apoptosis facilitator) // 12p13-p12 // 793	BCL2L14	NM_030766	0.0037691	2.26634
NR_002713 // NPY6R // neuropeptide Y receptor Y6 (pseudogene) // 5q31 // 4888 //	NPY6R	NR_002713	0.0429642	2.26407
NM_001114086 // CLIC5 // chloride intracellular channel 5 // 6p12.3 // 53405 ///	CLIC5	NM_001114086	0.0269601	2.25433
NM_003645 // SLC27A2 // solute carrier family 27 (fatty acid transporter), membe	SLC27A2	NM_003645	0.040906	2.2539
NM_001136050 // DHRS1 // dehydrogenase/reductase (SDR family) member 1 // 14q12	DHRS1	NM_001136050	0.000608529	2.23931
NM_002164 // IDO1 // indoleamine 2,3-dioxygenase 1 // 8p12-p11 // 3620 /// ENST0	IDO1	NM_002164	0.00532092	2.2314
NM_001171192 // GDPD2 // glycerophosphodiester phosphodiesterase domain containi	GDPD2	NM_001171192	0.0455387	2.23073
NM_016445 // PLEK2 // pleckstrin 2 // 14q23.3 // 26499 /// ENST00000216446 // PL	PLEK2	NM_016445	0.0184048	2.22972
NR_033122 // PDZD3 // PDZ domain containing 3 // 11q23.3 // 79849 /// NM_0011684	PDZD3	NR_033122	0.0104609	2.2269
NM_000932 // PLCB3 // phospholipase C, beta 3 (phosphatidylinositol-specific) //	PLCB3	NM_000932	0.01393	2.22018
NM_018235 // CNDP2 // CNDP dipeptidase 2 (metallopeptidase M20 family) // 18q22.	CNDP2	NM_018235	0.000958173	2.20566
NM_032562 // PLA2G12B // phospholipase A2, group XIIB // 10q22.1 // 84647 /// EN	PLA2G12B	NM_032562	0.0420214	2.20423
NM_021080 // DAB1 // disabled homolog 1 (Drosophila) // 1p32-p31 // 1600 /// ENS	DAB1	NM_021080	0.04076	2.20106
NM_001710 // CFB // complement factor B // 6p21.3 // 629 /// ENST00000425368 //	CFB	NM_001710	0.00181667	2.19954
NM_183240 // TMEM37 // transmembrane protein 37 // 2q14.2 // 140738 /// ENST0000	TMEM37	NM_183240	0.0487149	2.19842
AK127847 // FLJ45950 // FLJ45950 protein // 11q24.3 // 399975	FLJ45950	AK127847	0.00195329	2.198
NM_001710 // CFB // complement factor B // 6p21.3 // 629 /// ENST00000417261 //	CFB	NM_001710	0.00220919	2.19758
NM_144590 // ANKRD22 // ankyrin repeat domain 22 // 10q23.31 // 118932 /// ENST0	ANKRD22	NM_144590	0.0445105	2.19752
NM_002067 // GNA11 // guanine nucleotide binding protein (G protein), alpha 11 (	GNA11	NM_002067	0.014093	2.19185
NM_006579 // EBP // emopamil binding protein (sterol isomerase) // Xp11.23-p11.2	EBP	NM_006579	0.0115147	2.18786
NM_014873 // LPGAT1 // lysophosphatidylglycerol acyltransferase 1 // 1q32 // 992	LPGAT1	NM_014873	0.000550666	2.18469
NM_030943 // AMN // amnionless homolog (mouse) // 14q32.3 // 81693 /// ENST00000	AMN	NM_030943	0.00168811	2.18289
NM_016548 // GOLM1 // golgi membrane protein 1 // 9q21.33 // 51280 /// NM_177937	GOLM1	NM_016548	0.0424472	2.18243
NM_032148 // SLC41A2 // solute carrier family 41, member 2 // 12q23.3 // 84102 /	SLC41A2	NM_032148	0.0301277	2.17752
NM_000949 // PRLR // prolactin receptor // 5p13.2 // 5618 /// ENST00000382002 //	PRLR	NM_000949	0.0313649	2.17608
NM_181642 // SPINT1 // serine peptidase inhibitor, Kunitz type 1 // 15q15.1 // 6	SPINT1	NM_181642	0.0361797	2.17498
NM_001113567 // C17orf76 // chromosome 17 open reading frame 76 // 17p11.2 // 38	C17orf76	NM_001113567	0.0248369	2.17219
NM_000355 // TCN2 // transcobalamin II // 22q12.2 // 6948 /// NM_001184726 // TC	TCN2	NM_000355	0.0233279	2.17134

Supplementary Table 1: Complete Table of Microaray Targets, in decending order of fold change difference

Gene Information	Gene Symbol	RefSeq	p-value (CC vs. UC)	Fold Change (CC vs. UC)
NM_015198 // COBL // cordon-bleu homolog (mouse) // 7p12.1 // 23242 /// ENST0000	COBL	NM_015198	0.0208672	2.1656
NM_024616 // C3orf52 // chromosome 3 open reading frame 52 // 3q13.2 // 79669 //	C3orf52	NM_024616	0.00881101	2.16302
NM_020469 // ABO // ABO blood group (transferase A, alpha 1-3-N-acetylgalactosam	ABO	NM_020469	0.00222828	2.16292
NM_030908 // OR2A4 // olfactory receptor, family 2, subfamily A, member 4 // 6q2	OR2A4	NM_030908	0.00568966	2.15894
NM_003980 // MAP7 // microtubule-associated protein 7 // 6q23.3 // 9053 /// NM_0	MAP7	NM_003980	0.0037529	2.15742
NM_017417 // GALNT8 // UDP-N-acetyl-alpha-D-galactosamine:polypeptide N-acetylga	GALNT8	NM_017417	0.013696	2.15417
NM_005410 // SEPP1 // selenoprotein P, plasma, 1 // 5q31 // 6414 /// NM_00108548	SEPP1	NM_005410	0.0133071	2.15347
NM_152573 // RASEF // RAS and EF-hand domain containing // 9q21.32 // 158158 ///	RASEF	NM_152573	0.0366785	2.15133
NM_006633 // IQGAP2 // IQ motif containing GTPase activating protein 2 // 5q13.3	IQGAP2	NM_006633	0.00969849	2.1509
NM_152550 // SH3RF2 // SH3 domain containing ring finger 2 // 5q32 // 153769 ///	SH3RF2	NM_152550	0.00614396	2.15072
NM_018686 // CMAS // cytidine monophosphate N-acetylneuraminic acid synthetase /	CMAS	NM_018686	0.0124234	2.14998
NM_025045 // BAIAP2L2 // BAI1-associated protein 2-like 2 // 22q13.1 // 80115 //	BAIAP2L2	NM_025045	0.0129162	2.14195
NM_001859 // SLC31A1 // solute carrier family 31 (copper transporters), member 1	SLC31A1	NM_001859	0.00838827	2.13821
NM_016614 // TDP2 // tyrosyl-DNA phosphodiesterase 2 // 6p22.3-p22.1 // 51567 //	TDP2	NM_016614	0.0246156	2.13573
NM_003848 // SUCLG2 // succinate-CoA ligase, GDP-forming, beta subunit // 3p14.1	SUCLG2	NM_003848	0.00569037	2.13077
NM_017904 // TTC22 // tetratricopeptide repeat domain 22 // 1p32.3 // 55001 ///	TTC22	NM_017904	0.0153126	2.12827
NM_003060 // SLC22A5 // solute carrier family 22 (organic cation/carnitine trans	SLC22A5	NM_003060	0.02024	2.12394
NM_002662 // PLD1 // phospholipase D1, phosphatidylcholine-specific // 3q26 // 5	PLD1	NM_002662	0.0135876	2.12113
NM_018964 // SLC37A1 // solute carrier family 37 (glycerol-3-phosphate transport	SLC37A1	NM_018964	0.0229039	2.12062
NM_001251 // CD68 // CD68 molecule // 17p13 // 968 /// NM_001040059 // CD68 // C	CD68	NM_001251	0.00105743	2.11575
NM_174941 // CD163L1 // CD163 molecule-like 1 // 12p13.3 // 283316 /// ENST00000	CD163L1	NM_174941	0.00407203	2.11396
NM_016029 // DHRS7 // dehydrogenase/reductase (SDR family) member 7 // 14q23.1 /	DHRS7	NM_016029	0.0124063	2.11159
NM_024101 // MLPH // melanophilin // 2q37.3 // 79083 /// NM_001042467 // MLPH //	MLPH	NM_024101	0.00197625	2.10533
NM_004670 // PAPSS2 // 3'-phosphoadenosine 5'-phosphosulfate synthase 2 // 10q24	PAPSS2	NM_004670	0.0403309	2.10272
AK172782 // GPAM // glycerol-3-phosphate acyltransferase, mitochondrial // 10q25	GPAM	AK172782	0.0314353	2.09633
NM_001142685 // ARHGAP32 // Rho GTPase activating protein 32 // 11q24.3 // 9743	ARHGAP32	NM_001142685	0.00415504	2.09203
NM_198495 // CTAGE4 // CTAGE family, member 4 // 7q35 // 100128553 /// NM_001145	CTAGE4	NM_198495	0.00141321	2.0906
ENST00000439698 // P4HA2 // prolyl 4-hydroxylase, alpha polypeptide II // 5q31 /	P4HA2	ENST00000439698	0.0142839	2.08741
NM_015020 // PHLPP2 // PH domain and leucine rich repeat protein phosphatase 2 /	PHLPP2	NM_015020	0.013905	2.08634
NM_004252 // SLC9A3R1 // solute carrier family 9 (sodium/hydrogen exchanger), me	SLC9A3R1	NM_004252	0.00776993	2.0857
NM_012243 // SLC35A3 // solute carrier family 35 (UDP-N-acetylglucosamine (UDP-G	SLC35A3	NM_012243	0.0307101	2.07986
NM_020184 // CNNM4 // cyclin M4 // 2q11 // 26504 /// ENST00000377075 // CNNM4 //	CNNM4	NM_020184	0.02685	2.07897
NM_001490 // GCNT1 // glucosaminyl (N-acetyl) transferase 1, core 2 // 9q13 // 2	GCNT1	NM_001490	0.00172819	2.07671
NM_003667 // LGR5 // leucine-rich repeat-containing G protein-coupled receptor 5	LGR5	NM_003667	0.0237574	2.07254
NM_001966 // EHHADH // enoyl-CoA, hydratase/3-hydroxyacyl CoA dehydrogenase // 3	EHHADH	NM_001966	0.0130422	2.07114

Supplementary Table 1: Complete Table of Microaray Targets, in decending order of fold change difference

Gene Information	Gene Symbol	RefSeq	p-value (CC vs. UC)	Fold Change (CC vs. UC)
NM_017726 // PPP1R14D // protein phosphatase 1, regulatory (inhibitor) subunit 1	PPP1R14D	NM_017726	0.0497008	2.07017
NM_006994 // BTN3A3 // butyrophilin, subfamily 3, member A3 // 6p21.3 // 10384 /	BTN3A3	NM_006994	0.00121808	2.06925
NM_001039724 // NOSTRIN // nitric oxide synthase trafficker // 2q31.1 // 115677	NOSTRIN	NM_001039724	0.00986343	2.06731
NR_026912 // ABHD11 // abhydrolase domain containing 11 // 7q11.23 // 83451 ///	ABHD11	NR_026912	0.000593971	2.05896
NM_001145206 // KIAA1671 // KIAA1671 // 22q11.23 // 85379 /// ENST00000358431 //	KIAA1671	NM_001145206	0.00446756	2.05612
NM_153345 // TMEM139 // transmembrane protein 139 // 7q34 // 135932 /// ENST0000	TMEM139	NM_153345	0.00505302	2.05293
NM_001164694 // IYD // iodotyrosine deiodinase // 6q25.1 // 389434 /// NM_203395	IYD	NM_001164694	0.022189	2.05208
NM_016472 // C14orf129 // chromosome 14 open reading frame 129 // 14q32.2 // 515	C14orf129	NM_016472	0.048055	2.04519
NM_001017402 // LAMB3 // laminin, beta 3 // 1q32 // 3914 /// NM_001127641 // LAM	LAMB3	NM_001017402	0.0267716	2.04174
NM_004999 // MYO6 // myosin VI // 6q13 // 4646 /// ENST00000369977 // MYO6 // my	MYO6	NM_004999	0.00369349	2.04095
NR_027244 // LOC151009 // hypothetical LOC151009 // 2q13 // 151009 /// NR_027244	LOC151009	NR_027244	0.0115721	2.04078
AB065085 // TOM1L1 // target of myb1 (chicken)-like 1 // 17q23.2 // 10040	TOM1L1	AB065085	0.04656	2.03713
NM_017750 // RETSAT // retinol saturase (all-trans-retinol 13,14-reductase) // 2	RETSAT	NM_017750	0.0184264	2.03345
NM_004721 // MAP3K13 // mitogen-activated protein kinase kinase kinase 13 // 3q2	MAP3K13	NM_004721	0.00937615	2.03148
NM_018677 // ACSS2 // acyl-CoA synthetase short-chain family member 2 // 20q11.2	ACSS2	NM_018677	0.0306269	2.02661
NM_014317 // PDSS1 // prenyl (decaprenyl) diphosphate synthase, subunit 1 // 10p	PDSS1	NM_014317	0.0365076	2.02171
NM_014498 // GOLIM4 // golgi integral membrane protein 4 // 3q26.2 // 27333 ///	GOLIM4	NM_014498	0.00240934	2.02056
NM_033429 // CALML4 // calmodulin-like 4 // 15q23 // 91860 /// NM_001031733 // C	CALML4	NM_033429	0.0419784	2.01981
NR_036751 // HSP90AA6P // heat shock protein 90kDa alpha (cytosolic), class A me	HSP90AA6P	NR_036751	0.0220954	2.01604
NM_012120 // CD2AP // CD2-associated protein // 6p12 // 23607 /// ENST0000035931	CD2AP	NM_012120	0.00502091	2.0122
NM_005536 // IMPA1 // inositol(myo)-1(or 4)-monophosphatase 1 // 8q21.13-q21.3 /	IMPA1	NM_005536	0.0194688	2.01203
NM_001153 // ANXA4 // annexin A4 // 2p13 // 307 /// ENST00000394295 // ANXA4 //	ANXA4	NM_001153	0.0255723	2.01151
NM_000147 // FUCA1 // fucosidase, alpha-L- 1, tissue // 1p34 // 2517 /// ENST000	FUCA1	NM_000147	0.00469253	2.0105
NM_003774 // GALNT4 // UDP-N-acetyl-alpha-D-galactosamine:polypeptide N-acetylga	GALNT4	NM_003774	0.00622316	2.00871
NM_001122890 // GGT6 // gamma-glutamyltransferase 6 // 17p13.2 // 124975 /// NM_	GGT6	NM_001122890	0.0328357	2.00627
NM_001164277 // SLC37A4 // solute carrier family 37 (glucose-6-phosphate transpo	SLC37A4	NM_001164277	0.0068184	2.00477
NM_001565 // CXCL10 // chemokine (C-X-C motif) ligand 10 // 4q21 // 3627 /// ENS	CXCL10	NM_001565	0.0468134	2.00368
NM_005030 // PLK1 // polo-like kinase 1 // 16p12.2 // 5347 /// ENST00000300093 /	PLK1	NM_005030	0.0109795	2.00251
NM_001012631 // IL32 // interleukin 32 // 16p13.3 // 9235 /// NM_004221 // IL32	IL32	NM_001012631	0.0214868	2.00238
NM_005309 // GPT // glutamic-pyruvate transaminase (alanine aminotransferase) //	GPT	NM_005309	0.0098254	2.00201
NM_005159 // ACTC1 // actin, alpha, cardiac muscle 1 // 15q11-q14 // 70 /// ENST	ACTC1	NM_005159	0.00451989	-2.00712
NM_130385 // MRVI1 // murine retrovirus integration site 1 homolog // 11p15 // 1	MRVI1	NM_130385	0.0186352	-2.00908
NR_003329 // SNORD116-14 // small nucleolar RNA, C/D box 116-14 // 15q11.2 // 10	SNORD116-14	NR_003329	0.00710694	-2.01066
NM_030751 // ZEB1 // zinc finger E-box binding homeobox 1 // 10p11.2 // 6935 ///	ZEB1	NM_030751	0.0190641	-2.01665
NM_001321 // CSRP2 // cysteine and glycine-rich protein 2 // 12q21.1 // 1466 ///	CSRP2	NM_001321	0.0130189	-2.01975

Supplementary Table 1: Complete Table of Microaray Targets, in decending order of fold change difference

Gene Information	Gene Symbol	RefSeq	p-value (CC vs. UC)	Fold Change (CC vs. UC)
NM_199460 // CACNA1C // calcium channel, voltage-dependent, L type, alpha 1C sub	CACNA1C	NM_199460	0.0164629	-2.03364
NM_007078 // LDB3 // LIM domain binding 3 // 10q22.3-q23.2 // 11155 /// NM_00117	LDB3	NM_007078	0.013344	-2.03636
ENST00000436525 // C15orf51 // dynamin 1 pseudogene // 15q26.3 // 196968	C15orf51	ENST00000436525	0.0479813	-2.04311
ENST00000436525 // C15orf51 // dynamin 1 pseudogene // 15q26.3 // 196968	C15orf51	ENST00000436525	0.0479813	-2.04311
NM_001042454 // TGFB1I1 // transforming growth factor beta 1 induced transcript	TGFB1I1	NM_001042454	0.0141045	-2.0503
NM_201266 // NRP2 // neuropilin 2 // 2q33.3 // 8828 /// NM_003872 // NRP2 // neu	NRP2	NM_201266	0.0231808	-2.05329
NM_014286 // NCS1 // neuronal calcium sensor 1 // 9q34 // 23413 /// NM_001128826	NCS1	NM_014286	0.0400809	-2.05571
NR_002960 // SNORA20 // small nucleolar RNA, H/ACA box 20 // 6q25.3 // 677806	SNORA20	NR_002960	0.0102255	-2.05618
NR_023343 // RNU4ATAC // RNA, U4atac small nuclear (U12-dependent splicing) // 2	RNU4ATAC	NR_023343	0.0114016	-2.05953
NM_003829 // MPDZ // multiple PDZ domain protein // 9p23 // 8777 /// ENST0000038	MPDZ	NM_003829	0.0230169	-2.06542
NM_182734 // PLCB1 // phospholipase C, beta 1 (phosphoinositide-specific) // 20p	PLCB1	NM_182734	0.0285626	-2.0675
NM_212482 // FN1 // fibronectin 1 // 2q34 // 2335 /// NM_002026 // FN1 // fibron	FN1	NM_212482	0.0289963	-2.06817
NM_001166292 // PTCH2 // patched 2 // 1p34.1 // 8643 /// ENST00000438067 // PTCH	PTCH2	NM_001166292	0.0155977	-2.06949
NM_001128310 // SPARCL1 // SPARC-like 1 (hevin) // 4q22.1 // 8404 /// NM_004684	SPARCL1	NM_001128310	0.0275433	-2.0695
NR_003332 // SNORD116-17 // small nucleolar RNA, C/D box 116-17 // 15q11.2 // 10	SNORD116-17	NR_003332	0.00123218	-2.07085
NR_003332 // SNORD116-17 // small nucleolar RNA, C/D box 116-17 // 15q11.2 // 10	SNORD116-17	NR_003332	0.00123218	-2.07085
NM_001390 // DTNA // dystrobrevin, alpha // 18q12 // 1837 /// NM_032975 // DTNA	DTNA	NM_001390	0.0140008	-2.07227
NM_172316 // MEIS2 // Meis homeobox 2 // 15q14 // 4212 /// NM_170677 // MEIS2 //	MEIS2	NM_172316	0.012629	-2.07482
NM_032801 // JAM3 // junctional adhesion molecule 3 // 11q25 // 83700 /// ENST00	JAM3	NM_032801	0.00375191	-2.08055
NM_001496 // GFRA3 // GDNF family receptor alpha 3 // 5q31.1-q31.3 // 2676 /// E	GFRA3	NM_001496	0.0143176	-2.08436
NM_003116 // SPAG4 // sperm associated antigen 4 // 20q11.21 // 6676 /// ENST000	SPAG4	NM_003116	0.0370178	-2.09743
NR_002754 // RNU5E // RNA, U5E small nuclear // 1p36.22 // 26829 /// M77839 // R	RNU5E	NR_002754	0.0153145	-2.10499
NM_000109 // DMD // dystrophin // Xp21.2 // 1756 /// NM_004010 // DMD // dystrop	DMD	NM_000109	0.0305823	-2.10535
NM_005725 // TSPAN2 // tetraspanin 2 // 1p13.2 // 10100 /// ENST00000369516 // T	TSPAN2	NM_005725	0.00484522	-2.10726
ENST00000436525 // C15orf51 // dynamin 1 pseudogene // 15q26.3 // 196968	C15orf51	ENST00000436525	0.0401346	-2.11861
NM_001190839 // MGP // matrix Gla protein // 12p12.3 // 4256 /// NM_000900 // MG	MGP	NM_001190839	0.0229696	-2.13146
NM_031442 // TMEM47 // transmembrane protein 47 // Xp11.4 // 83604 /// ENST00000	TMEM47	NM_031442	0.0162367	-2.16059
NM_002776 // KLK10 // kallikrein-related peptidase 10 // 19q13 // 5655 /// NM_14	KLK10	NM_002776	0.0131782	-2.16442
NM_134269 // SMTN // smoothelin // 22q12.2 // 6525 /// NM_134270 // SMTN // smoo	SMTN	NM_134269	0.0278447	-2.16615
NM_002742 // PRKD1 // protein kinase D1 // 14q11 // 5587 /// ENST00000331968 //	PRKD1	NM_002742	0.0208525	-2.17797
NM_001001396 // ATP2B4 // ATPase, Ca++ transporting, plasma membrane 4 // 1q32.1	ATP2B4	NM_001001396	0.0372252	-2.18014
NM_005451 // PDLIM7 // PDZ and LIM domain 7 (enigma) // 5q35.3 // 9260 /// NM_20	PDLIM7	NM_005451	0.00654348	-2.18595
NR_002952 // SNORA9 // small nucleolar RNA, H/ACA box 9 // 7p13 // 677798 /// AK	SNORA9	NR_002952	0.0244704	-2.19918
NM_003069 // SMARCA1 // SWI/SNF related, matrix associated, actin dependent regu	SMARCA1	NM_003069	0.00571381	-2.2109
NR_003330 // SNORD116-15 // small nucleolar RNA, C/D box 116-15 // 15q11.2 // 10	SNORD116-15	NR_003330	6.72E-05	-2.21218

Supplementary Table 1: Complete Table of Microaray Targets, in decending order of fold change difference

Gene Information	Gene Symbol	RefSeq	p-value (CC vs. UC)	Fold Change (CC vs. UC)
NM_002398 // MEIS1 // Meis homeobox 1 // 2p14 // 4211 /// ENST00000272369 // MEI	MEIS1	NM_002398	0.0208728	-2.21341
ENST00000436525 // C15orf51 // dynamin 1 pseudogene // 15q26.3 // 196968	C15orf51	ENST00000436525	0.0297132	-2.22015
ENST00000436525 // C15orf51 // dynamin 1 pseudogene // 15q26.3 // 196968	C15orf51	ENST00000436525	0.0297132	-2.22015
NM_003734 // AOC3 // amine oxidase, copper containing 3 (vascular adhesion prote	AOC3	NM_003734	0.0151647	-2.22019
AF391113 // C21orf70 // chromosome 21 open reading frame 70 // 21q22.3 // 85395	C21orf70	AF391113	0.00109586	-2.22308
NM_001937 // DPT // dermatopontin // 1q12-q23 // 1805 /// ENST00000367817 // DPT	DPT	NM_001937	0.0379186	-2.22359
NM_012232 // PTRF // polymerase I and transcript release factor // 17q21.2 // 28	PTRF	NM_012232	0.0194925	-2.23107
NM_024605 // ARHGAP10 // Rho GTPase activating protein 10 // 4q31.23 // 79658 //	ARHGAP10	NM_024605	0.00832518	-2.23204
NM_022117 // TSPYL2 // TSPY-like 2 // Xp11.2 // 64061 /// ENST00000375442 // TSP	TSPYL2	NM_022117	0.0134024	-2.23502
NM_005100 // AKAP12 // A kinase (PRKA) anchor protein 12 // 6q24-q25 // 9590 ///	AKAP12	NM_005100	0.0357306	-2.24089
AY423733 // DDR2 // discoidin domain receptor tyrosine kinase 2 // 1q23.3 // 492	DDR2	AY423733	0.0358613	-2.2447
NM_153703 // PODN // podocan // 1p32.3 // 127435 /// ENST00000312553 // PODN //	PODN	NM_153703	0.0277365	-2.26923
NM_004370 // COL12A1 // collagen, type XII, alpha 1 // 6q12-q13 // 1303 /// NM_0	COL12A1	NM_004370	0.0499701	-2.27002
NM_004137 // KCNMB1 // potassium large conductance calcium-activated channel, su	KCNMB1	NM_004137	0.0277682	-2.27584
NM_014575 // SCHIP1 // schwannomin interacting protein 1 // 3q25.32-q25.33 // 29	SCHIP1	NM_014575	0.00470657	-2.28272
NM_001753 // CAV1 // caveolin 1, caveolae protein, 22kDa // 7q31.1 // 857 /// NM	CAV1	NM_001753	0.0368534	-2.29054
NM_002338 // LSAMP // limbic system-associated membrane protein // 3q13.2-q21 //	LSAMP	NM_002338	0.0456749	-2.30408
NM_058229 // FBXO32 // F-box protein 32 // 8q24.13 // 114907 /// NM_148177 // FB	FBXO32	NM_058229	0.0422526	-2.30763
NM_006765 // TUSC3 // tumor suppressor candidate 3 // 8p22 // 7991 /// NM_178234	TUSC3	NM_006765	0.00173576	-2.32217
NM_015687 // FILIP1 // filamin A interacting protein 1 // 6q14.1 // 27145 /// EN	FILIP1	NM_015687	0.0158717	-2.32321
NM_006080 // SEMA3A // sema domain, immunoglobulin domain (Ig), short basic doma	SEMA3A	NM_006080	0.0142131	-2.32699
NM_000922 // PDE3B // phosphodiesterase 3B, cGMP-inhibited // 11p15.1 // 5140 //	PDE3B	NM_000922	0.00420057	-2.33135
NM_000722 // CACNA2D1 // calcium channel, voltage-dependent, alpha 2/delta subun	CACNA2D1	NM_000722	0.0107345	-2.33411
NM_001197294 // DPYSL3 // dihydropyrimidinase-like 3 // 5q32 // 1809 /// NM_0013	DPYSL3	NM_001197294	0.0231385	-2.33517
NM_172311 // STON1-GTF2A1L // STON1-GTF2A1L readthrough // 2p16.3 // 286749 ///	STON1-GTF2A1	NM_172311	0.0264382	-2.33729
NM_000857 // GUCY1B3 // guanylate cyclase 1, soluble, beta 3 // 4q31.3-q33 // 29	GUCY1B3	NM_000857	0.0141507	-2.34285
NR_033662 // CSF3 // colony stimulating factor 3 (granulocyte) // 17q11.2-q12 //	CSF3	NR_033662	0.036854	-2.35397
NM_001706 // BCL6 // B-cell CLL/lymphoma 6 // 3q27 // 604 /// NM_001130845 // BC	BCL6	NM_001706	0.0395014	-2.37213
NM_014112 // TRPS1 // trichorhinophalangeal syndrome I // 8q24.12 // 7227 /// EN	TRPS1	NM_014112	0.021813	-2.37338
NM_003275 // TMOD1 // tropomodulin 1 // 9q22.3 // 7111 /// NM_001166116 // TMOD1	TMOD1	NM_003275	0.00926909	-2.39163
NM_004040 // RHOB // ras homolog gene family, member B // 2p24 // 388 /// ENST00	RHOB	NM_004040	0.00209611	-2.39166
NM_007281 // SCRG1 // stimulator of chondrogenesis 1 // 4q34.1 // 11341 /// ENST	SCRG1	NM_007281	0.0449505	-2.42771
NM_053025 // MYLK // myosin light chain kinase // 3q21 // 4638 /// NM_053026 //	MYLK	NM_053025	0.0334323	-2.44896
NM_133646 // ZAK // sterile alpha motif and leucine zipper containing kinase AZK	ZAK	NM_133646	0.0101002	-2.45225
NM_001123364 // C6orf186 // chromosome 6 open reading frame 186 // 6q21 // 72846	C6orf186	NM_001123364	0.0338175	-2.45305

Supplementary Table 1: Complete Table of Microaray Targets, in decending order of fold change difference

Gene Information	Gene Symbol	RefSeq	p-value (CC vs. UC)	Fold Change (CC vs. UC)
NM_005909 // MAP1B // microtubule-associated protein 1B // 5q13 // 4131 /// ENST	MAP1B	NM_005909	0.00199713	-2.45363
NM_001136191 // KANK2 // KN motif and ankyrin repeat domains 2 // 19p13.2 // 259	KANK2	NM_001136191	0.00418	-2.45823
NR_002836 // PGM5P2 // phosphoglucomutase 5 pseudogene 2 // 9q12 // 595135 /// N	PGM5P2	NR_002836	0.0106051	-2.46207
NM_006988 // ADAMTS1 // ADAM metallopeptidase with thrombospondin type 1 motif,	ADAMTS1	NM_006988	0.0212926	-2.47602
NM_001897 // CSPG4 // chondroitin sulfate proteoglycan 4 // 15q24.2 // 1464 ///	CSPG4	NM_001897	0.000233664	-2.47738
NM_012134 // LMOD1 // leiomodin 1 (smooth muscle) // 1q32 // 25802 /// ENST00000	LMOD1	NM_012134	0.0254164	-2.48821
NM_000856 // GUCY1A3 // guanylate cyclase 1, soluble, alpha 3 // 4q31.3-q33|4q31	GUCY1A3	NM_000856	0.0154068	-2.49669
NR_002196 // H19 // H19, imprinted maternally expressed transcript (non-protein	H19	NR_002196	0.0422207	-2.49895
NM_002667 // PLN // phospholamban // 6q22.1 // 5350 /// ENST00000357525 // PLN /	PLN	NM_002667	0.0458219	-2.50528
NM_004078 // CSRP1 // cysteine and glycine-rich protein 1 // 1q32 // 1465 /// NM	CSRP1	NM_004078	0.0389579	-2.51599
NM_001141945 // ACTA2 // actin, alpha 2, smooth muscle, aorta // 10q23.3 // 59 /	ACTA2	NM_001141945	0.00367966	-2.51621
NM_002986 // CCL11 // chemokine (C-C motif) ligand 11 // 17q21.1-q21.2 // 6356 /	CCL11	NM_002986	0.0132628	-2.5178
NM_033138 // CALD1 // caldesmon 1 // 7q33 // 800 /// NM_033157 // CALD1 // calde	CALD1	NM_033138	0.0229067	-2.51869
NM_001164836 // FXYD6 // FXYD domain containing ion transport regulator 6 // 11q	FXYD6	NM_001164836	0.0202065	-2.53004
NM_003725 // HSD17B6 // hydroxysteroid (17-beta) dehydrogenase 6 homolog (mouse)	HSD17B6	NM_003725	0.0196889	-2.54527
NM_001146312 // MYOCD // myocardin // 17p11.2 // 93649 /// NM_153604 // MYOCD //	MYOCD	NM_001146312	0.0298805	-2.59465
NM_015225 // PRUNE2 // prune homolog 2 (Drosophila) // 9q21.2 // 158471 /// AB53	PRUNE2	NM_015225	0.0217217	-2.59492
NM_001168278 // WWTR1 // WW domain containing transcription regulator 1 // 3q23-	WWTR1	NM_001168278	0.014475	-2.60243
NM_001008711 // RBPMS // RNA binding protein with multiple splicing // 8p12 // 1	RBPMS	NM_001008711	0.00600769	-2.60406
NM_001014796 // DDR2 // discoidin domain receptor tyrosine kinase 2 // 1q23.3 //	DDR2	NM_001014796	0.00523497	-2.61121
NM_018640 // LMO3 // LIM domain only 3 (rhombotin-like 2) // 12p12.3 // 55885 //	LMO3	NM_018640	0.042971	-2.63105
NR_002836 // PGM5P2 // phosphoglucomutase 5 pseudogene 2 // 9q12 // 595135 /// N	PGM5P2	NR_002836	0.00678244	-2.64929
NM_021914 // CFL2 // cofilin 2 (muscle) // 14q12 // 1073 /// NM_138638 // CFL2 /	CFL2	NM_021914	0.0261349	-2.65343
NM_016277 // RAB23 // RAB23, member RAS oncogene family // 6p11 // 51715 /// NM_	RAB23	NM_016277	0.035448	-2.66122
NM_145234 // CHRDL1 // chordin-like 1 // Xq23 // 91851 /// NM_001143981 // CHRDL	CHRDL1	NM_145234	0.00265317	-2.67563
NM_001134439 // PHLDB2 // pleckstrin homology-like domain, family B, member 2 //	PHLDB2	NM_001134439	0.0258326	-2.67775
NM_006832 // FERMT2 // fermitin family member 2 // 14q22.1 // 10979 /// NM_00113	FERMT2	NM_006832	0.0205617	-2.7145
NM_001128205 // SULF1 // sulfatase 1 // 8q13.1 // 23213 /// NM_015170 // SULF1 /	SULF1	NM_001128205	0.0335496	-2.73234
NM_194272 // RBPMS2 // RNA binding protein with multiple splicing 2 // 15q22.31	RBPMS2	NM_194272	0.012053	-2.74286
NM_014476 // PDLIM3 // PDZ and LIM domain 3 // 4q35 // 27295 /// NM_001114107 //	PDLIM3	NM_014476	0.0110612	-2.7574
NM_015886 // PI15 // peptidase inhibitor 15 // 8q21.11 // 51050 /// ENST00000260	PI15	NM_015886	0.0312943	-2.78937
NM_003289 // TPM2 // tropomyosin 2 (beta) // 9p13 // 7169 /// NM_213674 // TPM2	TPM2	NM_003289	0.0272347	-2.80338
NM_001458 // FLNC // filamin C, gamma // 7q32-q35 // 2318 /// NM_001127487 // FL	FLNC	NM_001458	0.0113027	-2.80588
NM_006097 // MYL9 // myosin, light chain 9, regulatory // 20q11.23 // 10398 ///	MYL9	NM_006097	0.0412118	-2.81849
NM_199460 // CACNA1C // calcium channel, voltage-dependent, L type, alpha 1C sub	CACNA1C	NM_199460	0.00694625	-2.83404

Supplementary Table 1: Complete Table of Microaray Targets, in decending order of fold change difference

Gene Information	Gene Symbol	RefSeq	p-value (CC vs. UC)	Fold Change (CC vs. UC)
NM_001232 // CASQ2 // calsequestrin 2 (cardiac muscle) // 1p13.3-p11 // 845 ///	CASQ2	NM_001232	0.0349505	-2.84886
NM_001193460 // MSRB3 // methionine sulfoxide reductase B3 // 12q14.3 // 253827	MSRB3	NM_001193460	0.0108076	-2.84899
NM_001456 // FLNA // filamin A, alpha // Xq28 // 2316 /// NM_001110556 // FLNA /	FLNA	NM_001456	0.0164878	-2.86026
NM_006366 // CAP2 // CAP, adenylate cyclase-associated protein, 2 (yeast) // 6p2	CAP2	NM_006366	0.00596997	-2.89059
NM_001031701 // NT5DC3 // 5'-nucleotidase domain containing 3 // 12q22-q23.1 //	NT5DC3	NM_001031701	0.0464686	-2.90347
NM_003999 // OSMR // oncostatin M receptor // 5p13.1 // 9180 /// NM_001168355 //	OSMR	NM_003999	0.0324297	-2.92605
NM_001885 // CRYAB // crystallin, alpha B // 11q22.3-q23.1 // 1410 /// ENST00000	CRYAB	NM_001885	0.0163674	-2.96044
NM_000517 // HBA2 // hemoglobin, alpha 2 // 16p13.3 // 3040 /// BC101846 // HBA1	HBA2	NM_000517	0.0195505	-3.10109
NM_000558 // HBA1 // hemoglobin, alpha 1 // 16p13.3 // 3039 /// BC101846 // HBA1	HBA1	NM_000558	0.0195505	-3.10109
NM_004282 // BAG2 // BCL2-associated athanogene 2 // 6p12.1-p11.2 // 9532 /// EN	BAG2	NM_004282	0.0108668	-3.11097
NM_022135 // POPDC2 // popeye domain containing 2 // 3q13.33 // 64091 /// ENST00	POPDC2	NM_022135	0.0219995	-3.1427
NM_001001522 // TAGLN // transgelin // 11q23.2 // 6876 /// NM_003186 // TAGLN //	TAGLN	NM_001001522	0.0148609	-3.35842
NM_212482 // FN1 // fibronectin 1 // 2q34 // 2335 /// NM_002026 // FN1 // fibron	FN1	NM_212482	0.00987492	-3.43741
NM_133477 // SYNPO2 // synaptopodin 2 // 4q26 // 171024 /// NM_001128933 // SYNP	SYNPO2	NM_133477	0.0241716	-3.56252
NM_000450 // SELE // selectin E // 1q22-q25 // 6401 /// ENST00000333360 // SELE	SELE	NM_000450	0.0460446	-3.56423
NR_029686 // MIR145 // microRNA 145 // 5q32 // 406937 /// NR_027180 // LOC728264	MIR145	NR_029686	0.0119026	-3.58867
NM_022648 // TNS1 // tensin 1 // 2q35-q36 // 7145 /// ENST00000171887 // TNS1 //	TNS1	NM_022648	0.00555851	-3.61273
NM_001615 // ACTG2 // actin, gamma 2, smooth muscle, enteric // 2p13.1 // 72 ///	ACTG2	NM_001615	0.0379131	-3.62826
NM_022844 // MYH11 // myosin, heavy chain 11, smooth muscle // 16p13.11 // 4629	MYH11	NM_022844	0.0240032	-3.66415
NM_002205 // ITGA5 // integrin, alpha 5 (fibronectin receptor, alpha polypeptide	ITGA5	NM_002205	0.0207749	-3.82521
NM_001299 // CNN1 // calponin 1, basic, smooth muscle // 19p13.2-p13.1 // 1264 /	CNN1	NM_001299	0.0413103	-3.84711
NM_001034954 // SORBS1 // sorbin and SH3 domain containing 1 // 10q23.33 // 1058	SORBS1	NM_001034954	0.00399907	-3.89048
NM_001927 // DES // desmin // 2q35 // 1674 /// ENST00000373960 // DES // desmin	DES	NM_001927	0.0268126	-3.90558
NM_144617 // HSPB6 // heat shock protein, alpha-crystallin-related, B6 // 19q13.	HSPB6	NM_144617	0.0145209	-3.90993
NM_015424 // CHRDL2 // chordin-like 2 // 11q14 // 25884 /// ENST00000263671 // C	CHRDL2	NM_015424	0.0247555	-4.23746
NM_000518 // HBB // hemoglobin, beta // 11p15.5 // 3043 /// ENST00000335295 // H	HBB	NM_000518	0.0255665	-4.3277
NM_002160 // TNC // tenascin C // 9q33 // 3371 /// ENST00000350763 // TNC // ten	TNC	NM_002160	0.0126641	-4.4403
NM_006198 // PCP4 // Purkinje cell protein 4 // 21q22.2 // 5121 /// ENST00000328	PCP4	NM_006198	0.0340302	-4.51736
